# Surge of neurophysiological coupling and connectivity of gamma oscillations in the dying human brain

**DOI:** 10.1073/pnas.2216268120

**Published:** 2023-05-01

**Authors:** Gang Xu, Temenuzhka Mihaylova, Duan Li, Fangyun Tian, Peter M. Farrehi, Jack M. Parent, George A. Mashour, Michael M. Wang, Jimo Borjigin

**Affiliations:** ^a^Department of Molecular and Integrative Physiology, University of Michigan School of Medicine, Ann Arbor, MI 48109; ^b^Department of Neurology, University of Michigan School of Medicine, Ann Arbor, MI 48109; ^c^Department of Internal Medicine-Cardiology, University of Michigan School of Medicine, Ann Arbor, MI 48109; ^d^Neuroscience Graduate Program, University of Michigan, Ann Arbor, MI 48109; ^e^Michigan Neuroscience Institute, University of Michigan, Ann Arbor, MI 48109; ^f^VA Ann Arbor Healthcare System, Ann Arbor, MI 48105; ^g^Department of Anesthesiology, University of Michigan School of Medicine, Ann Arbor, MI 48109; ^h^Center for Consciousness Science, University of Michigan, Ann Arbor, MI 48109

**Keywords:** global hypoxia, gamma oscillations, cross-frequency coupling, functional connectivity, directed connectivity

## Abstract

The brain is assumed to be hypoactive during cardiac arrest. However, animal models of cardiac and respiratory arrest demonstrate a surge of gamma oscillations and functional connectivity. To investigate whether these preclinical findings translate to humans, we analyzed electroencephalogram and electrocardiogram signals in four comatose dying patients before and after the withdrawal of ventilatory support. Two of the four patients exhibited a rapid and marked surge of gamma power, surge of cross-frequency coupling of gamma waves with slower oscillations, and increased interhemispheric functional and directed connectivity in gamma bands. High-frequency oscillations paralleled the activation of beta/gamma cross-frequency coupling within the somatosensory cortices. Importantly, both patients displayed surges of functional and directed connectivity at multiple frequency bands within the posterior cortical “hot zone,” a region postulated to be critical for conscious processing. This gamma activity was stimulated by global hypoxia and surged further as cardiac conditions deteriorated in the dying patients. These data demonstrate that the surge of gamma power and connectivity observed in animal models of cardiac arrest can be observed in select patients during the process of dying.

## Significance

Is it possible for the human brain to be activated by the dying process? We addressed this issue by analyzing the electroencephalograms (EEG) of four dying patients before and after the clinical withdrawal of their ventilatory support and found that the resultant global hypoxia markedly stimulated gamma activities in two of the patients. The surge of gamma connectivity was both local, within the temporo–parieto–occipital (TPO) junctions, and global between the TPO zones and the contralateral prefrontal areas. While the mechanisms and physiological significance of these findings remain to be fully explored, these data demonstrate that the dying brain can still be active. They also suggest the need to reevaluate role of the brain during cardiac arrest.

Brain function around the time of cardiac arrest is poorly understood. While the loss of *overt* consciousness is invariably associated with cardiac arrest, it is unclear whether patients can possess *covert* consciousness during the dying process. Near-death experiences (NDE) (1), reported to be highly lucid, “realer than real,” and common to people from diverse cultural and religious backgrounds (2, 3), have been described by 10 to 20% of cardiac arrest survivors (2–4). Indeed, it has been claimed (3, 5) that these episodes occur during clinical death even during electrical quiescence of the electroencephalogram (EEG). NDE represent a biological paradox that challenges our fundamental understanding of the dying brain, which is widely believed to be nonfunctioning under such conditions.

Elevation of high-frequency oscillations, a candidate marker of consciousness (6), has been reported previously in patients dying from critical illnesses (7–9). In healthy animals, our group reported that sudden termination of cardiac function or acute asphyxia stimulated high levels of gamma activities, including a global increase of functional and directed connectivity in gamma oscillations (10, 11). To date, however, no studies have reported neural correlates in dying humans that might account for the subjective percepts reported by near-death experiencers (1, 3, 12). The goal of this study was to identify neural correlates of the dying process.

Visual consciousness is classically associated with two distinct streams: the ventral stream coursing through the occipitotemporal (OT) junction that mediates visual object recognition, and the dorsal stream coursing through the occipitoparietal (OP) junction that serves nonconscious visually guided actions (13). A part of the dorsal stream also projects to the medial temporal lobe flowing through the temporoparietal (TP) junction mediating visuospatial processing (13). Importantly, visual sensations can be elicited by the stimulation of both occipital and parietal cortices in the absence of an external visual stimulus (14), and both temporal and parietal cortices function as early gatekeepers of perceptual awareness (15). Thus, activation of the temporo–parietal–occipital (TPO) junctions provides a key gateway for visuospatial processing in normal human brain (13). Critically, during dreaming, high-frequency activity within posterior cortical areas has been shown to predict perceptual content experienced by healthy subjects (16). Because this posterior cortical zone activated in dreaming (16) largely overlaps with that identified in waking (13), the TPO junctions are considered a “hot zone” for the neural correlates of consciousness (6). Internal perception of bright light or familiar faces reported by survivors of clinical death (3) suggests a preserved capacity in the dying brain to process internally generated vision. It remains to be determined if the posterior cortical regions are activated in the dying human brain.

These findings prompted us to investigate the neural activity of the brain in the dying patients before and after clinical withdrawal of ventilatory support. Specifically, we examined EEG signals, by applying the computational tools used in our previous study of dying animals (10), with a focus on the following features: temporal dynamics of EEG power, local and long-range phase-amplitude coupling between low- and high-frequency oscillations, and functional and directed cortical connectivity across all frequency bands. All analyses were conducted with close attention to parallel changes of electrocardiogram (ECG) signals, as reported previously (11).

## Results

### Patients.

This was a retrospective study of patients who died in the neurointensive care unit (NICU) at Michigan Medicine, University of Michigan since 2014. The study was approved by the Internal Review Board at the University of Michigan. We identified four patients (*SI Appendix,* Table S1) who passed away while undergoing EEG monitoring due to cardiac arrest-induced anoxic injury (Pt1, Pt2, Pt4) and extensive brain hemorrhage (Pt3). Three of the patients had preexisting cardiac conditions (long-QT syndrome for Pt1, heart transplant for Pt2, advanced coronary artery disease for Pt4), and two were diagnosed with previous seizures (Pt1) or in-hospital status epilepticus (Pt3). During the last 24 h, however, neither Pt1 nor Pt3 had detectable seizure activities, as confirmed on EEG by a seizure specialist. In the NICU, three of the four patients received one or more defibrillation due to ventricular tachycardia/fibrillation (3× for Pt1), ventricular tachycardia/fibrillation and pulseless electrical activity (3× for Pt2), and pulseless electrical activity (1× for Pt4). All patients were treated with hypothermia after resuscitation. All four patients were comatose at baseline (S1) with no evidence of voluntary behavior or any overt consciousness during the last 24 h of their lives; their Glasgow Coma Scale (GCS) scores were of 3 (Pt1, Pt2, and Pt4) and 4 (Pt3). Due to poor neurological prognosis and upon approval of the patients’ family members, life support was ultimately withdrawn from all four comatose patients. For Pt1, the external atrial pacemaker was turned off 273 seconds after the withdrawal of ventilatory support. Prior to the EEG analysis, the dying stages were defined in accordance with the changes of both ECG and EEG features. Baseline periods (stage 1, or S1) were chosen within the last few hours of patients’ life while they were receiving full life support, including ventilation. Stage 2 (S2) represents the period from the time of ventilator removal until the acute EEG suppression seen in all patients (*SI Appendix*, Fig. S1). Subsequent stages (S3-end) were defined according to the ECG features surveyed using the electrocardiomatrix (ECM) technique invented in the Borjigin laboratory (17) (*SI Appendix*, Fig. S1 and Table S2). All four patients exhibited low heartrate variability (HRV) at baseline (S1) (for SDNN: Pt1 > Pt3 > Pt4 > Pt2; *SI Appendix*, Table S2).

### Rise of Absolute EEG Power at Gamma Frequency Bands at Near-Death.

**Fig. 1.**
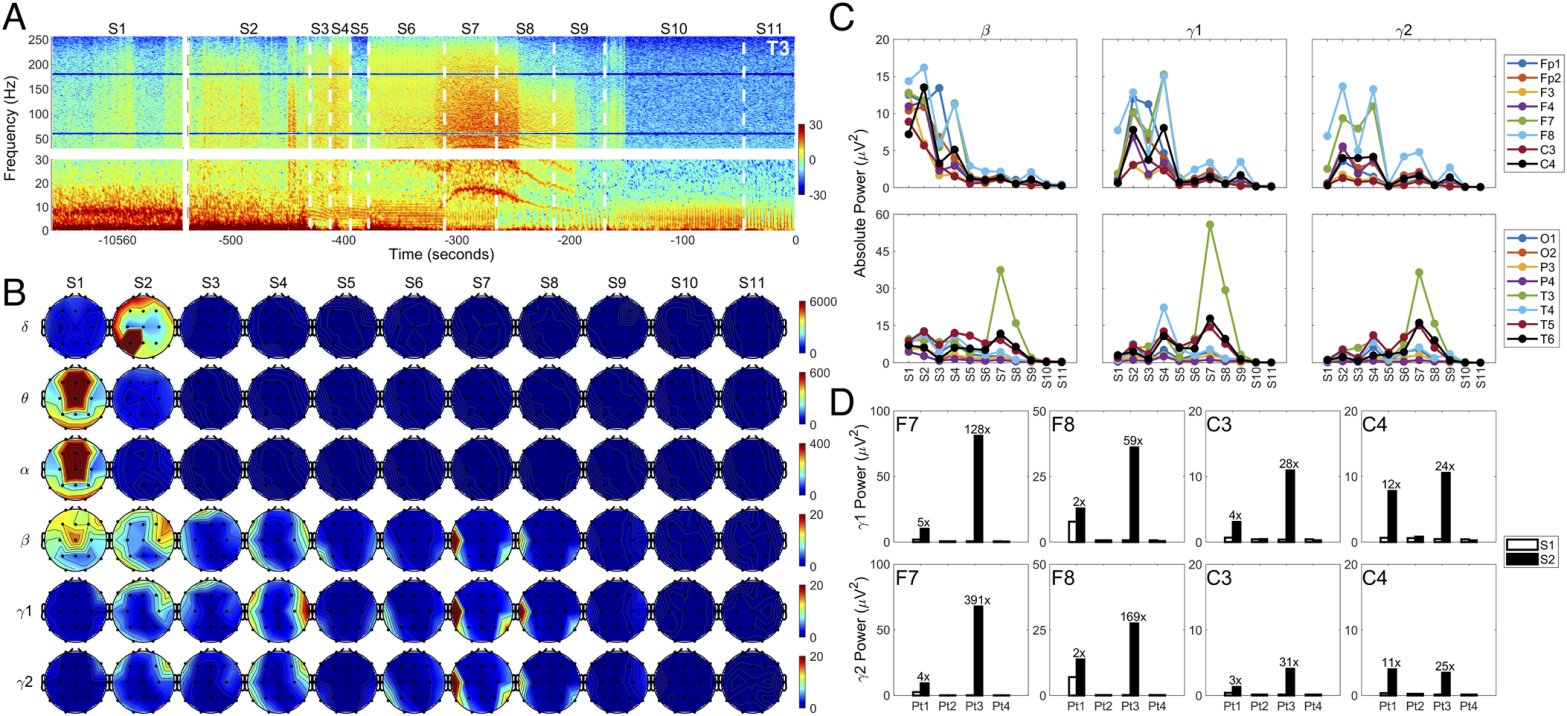
Global hypoxia-induced surge of high-frequency oscillations in the brain of dying patients. (*A*) Absolute power of left anterior-mid temporal lobe (T3) before (S1) and after (S2 to S11) the withdrawal of ventilatory support in Pt1. The power spectrogram was presented in two separate parts (*Bottom*: 0 to 30 Hz; *Top*: 30 to 256 Hz) to highlight potentially unique features in slow waves (delta-beta). (*B*) Spatial and temporal dynamics of absolute power at baseline (S1) and at near-death stages (S2 to S11) in six frequency bands: delta (0 to 4 Hz), theta (4 to 8 Hz), alpha (8 to 13 Hz), beta (13 to 25 Hz), gamma1 (25 to 55 Hz), and gamma2 (80 to 150 Hz) in Pt1. (*C*) Temporal changes of beta, gamma1, and gamma2 power in 16 EEG loci (excluding the midline areas covered by Fz, Cz, and Pz electrodes) in Pt1. (*D*) Gamma power in four cortical regions (F7, F8, C3, and C4) in the four patients at baseline (S1) and after the termination of breathing support in S2.

In the left anterior-mid temporal lobe (T3) of Pt1, gamma power (>25 Hz) showed a marked surge at near-death, increasing within seconds after ventilator removal in S2 and remaining visually detectable until the early period in S10 (Fig. 1*A*). Elevation of absolute EEG power in beta (15 to 25 Hz), gamma1 (25 to 55 Hz) and gamma2 (80 to 150 Hz) bands across the dying brain was evident in S2 in frontal and central areas for both Pt1 (Fig. 1*B*) and Pt3 (*SI Appendix*, Fig. S2*b*), while they were undetectable in Pt2 and Pt4 (*SI Appendix*, Fig. S2 *A* and *C*). Right (in S4) and left (in S7, S8) temporal lobes showed further increase of gamma power in the later dying stages in Pt1 (Fig. 1*B*). Quantification of temporal distribution of beta, gamma1, and gamma2 power across all monitored electrodes (Fig. 1*C*) confirmed the visual inspection (Fig. 1*B*) for Pt1: somatosensory cortices (SSCs; C3 and C4) as well as the right dorsolateral prefrontal cortex (DLPFC; F4), nearly silent in S1, showed a marked surge in gamma power in S2. The rise in gamma power was also seen in the left and right ventrolateral prefrontal cortex (VLPFC; F7, F8) as well as in the SSCs of both Pt1 (Fig. 1 *B*–*D*) and Pt3 (Fig. 1*D* and *SI Appendix*, Fig. S2*B*) with the increase ranging from 2- to 391-fold in S2 compared to S1 (Fig. 1*D*). Pt2 and Pt4 showed absence of gamma power increase at near-death (Fig. 1*D* and *SI Appendix*, Fig. S2 *A*–*C*). Therefore, we focused all our subsequent analyses on Pt1 and Pt3.

### Elevated Phase-Amplitude Coupling of Gamma Oscillations at Near-Death.

**Fig. 2.**
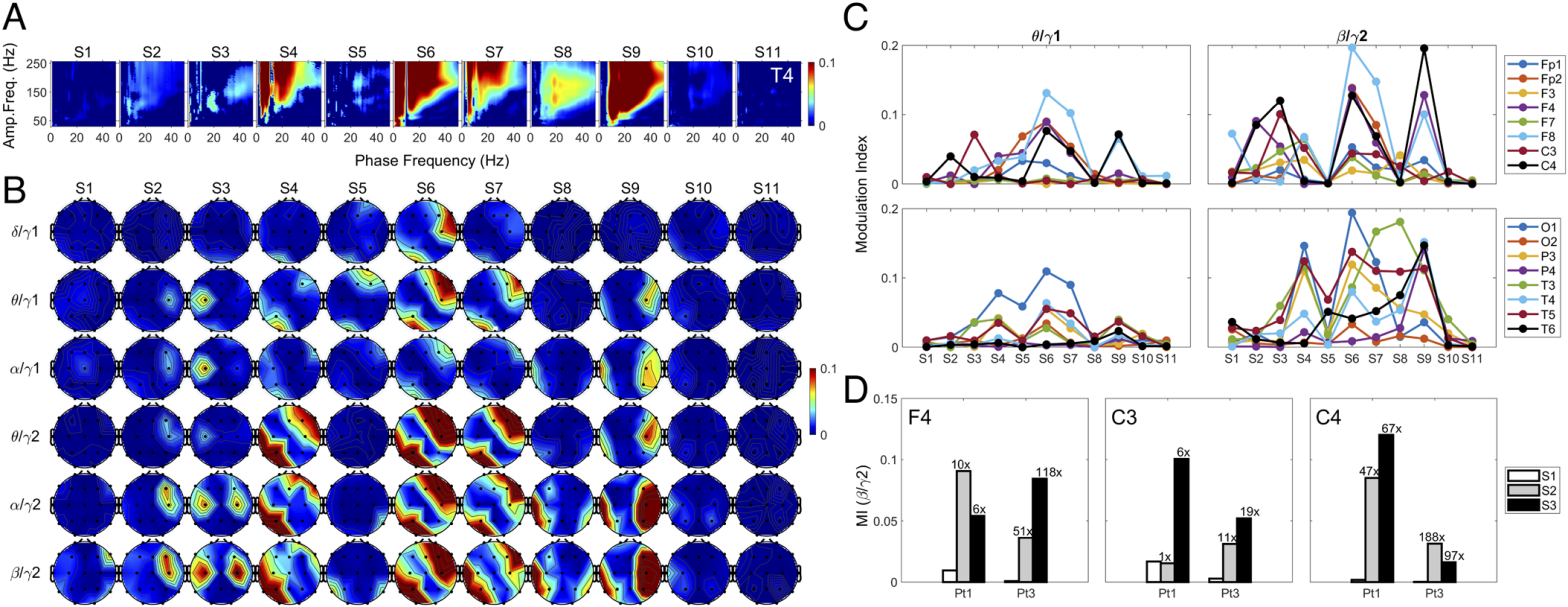
Hypoxia-induced surge of PAC in the dying brain. (*A*) Cross-frequency coupling between the phase of lower frequency bands (0 to 50 Hz) and amplitude of higher frequency waves (30 to 250 Hz) in the right anterior-mid temporal lobe (T4) in Pt1. (*B*) Spatial and temporal distribution of phase-amplitude coupling (PAC) between gamma (gamma1 and gamma2) and lower frequency (delta, theta, alpha, and beta) bands in Pt1 at baseline (S1) and near-death (S2 to S11). (*C*) Temporal changes of theta/gamma1 and beta/gamma2 coupling in Pt1 in 16 cortical regions at near-death. (*D*) Beta-gamma2 PAC in somatosensory cortices (C3 and C4) and right dorsal lateral prefrontal cortex (F4) in both Pt1 and Pt3 at baseline (S1) and during the early dying phase (S2, S3).

In the right anterior-mid temporal lobe (T4) of Pt1, the amplitude of gamma oscillations (>50 Hz) showed intense coupling to the phase of lower frequency bands (<50 Hz), whose coupling strength varied dynamically across the dying stages (Fig. 2*A*). Coupling was undetectable in S1 but showed marked increases in S4, S6, S7, and S9. In S6 and S9, extensive coupling was found between the phase of frequency bands (8 to 40 Hz) and amplitude of all frequency bands above 50 Hz. The frequency of coupled gamma oscillations peaked above 150 Hz. In this study, we restricted our focus on the coupling between the amplitude of gamma1 (25 to 55 Hz), gamma2 (80 to 150 Hz) and the phase of lower frequency bands (theta, alpha, and beta). The phase-amplitude coupling (PAC) was nearly absent at baseline (S1) for any of the frequency combinations (Fig. 2*B*) across the brain for Pt1. It became evident as early as in S2 for all displayed PAC types, except delta/gamma1 PAC (Fig. 2*B*). Interestingly, while PAC of all types rose only on the right SSC (C4) and right DLPFC (F4) in S2, the further rise of PAC in S3 was detected only in the left SSC (C3; Fig. 2*B*) for gamma1-associated PAC (theta/gamma1 and alpha/gamma1). For gamma2-associated PAC coupling (alpha/gamma2 and beta/gamma2), elevated coupling was expanded to both hemispheres in S3 that included the left SSC (C3), as well as the right DLPFC (F4) and right SSC (C4) (Fig. 2 *B* and *C*). During the periods when the external atrial pacemaker was triggered (S4, S6, S7) by the persistent decline of heart rate in S3, high levels of PAC clustered in the right anterior (Fp2, F8, F4, C4) and left posterior (T3, T5, P3, O1) areas for theta/gamma2, alpha/gamma2, and beta/gamma2 PACs (Fig. 2*B*). In S8 and S9 when the atrial pacemaker was off, PAC was detected mainly in the left temporal (T3 and T5 in S8) and right frontal, central, parietal, and temporal lobes (F4, F8, C4, T4, T6, P4 in S9) (Fig. 2 *B* and *C*). The surge of beta/gamma2 PAC, ranging 1- to 188-fold over S1 in S2 or S3, was detected in the right DLPFC (F4) and SSCs in both Pt1 (Fig. 2 *C* and *D*) and Pt3 (Fig. 2*D* and *SI Appendix*, Fig. S3*B*). Phase-amplitude coupling between gamma2 and lower frequency bands was absent in Pt2 (*SI Appendix*, Fig. S3*A*) and largely undetectable in Pt4 (*SI Appendix*, Fig. S3*C*).

### Increase of Cross-Regional Phase-Amplitude Coupling at Near-Death.

**Fig. 3.**
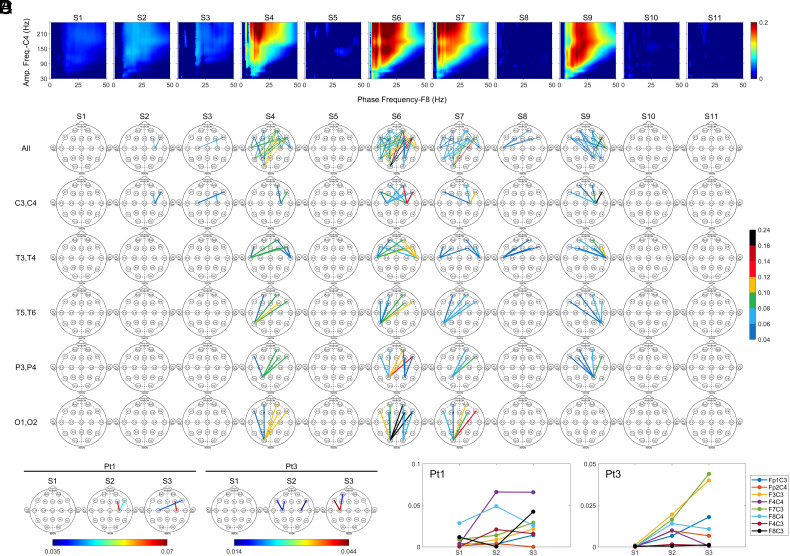
Surge of crPAC in the dying brain. (*A*) crPAC of gamma oscillations (30 to 250 Hz) in the right somatosensory cortex (C4) with the lower frequency (1 to 50 Hz) waves in the right ventrolateral prefrontal cortex (F8) displayed for Pt1. (*B*) Spatial and temporal dynamics of crPAC was displayed between amplitude of gamma2 wave in the posterior brain (C3, C4, T3, T4, T5, T6, P3, P4, O1 and O2) and phase of beta wave in the prefrontal lobes (Fp1, F7, F3, Fp2, F4, F8) in Pt1. The color bar indicates the strength of the beta/gamma2 coupling. (*C*) crPAC during the initial phase of the near-death states (S2 and S3) is readily detectable only between the somatosensory cortices (C3 and C4) and the prefrontal (Fp1, F7, F3, F4, F8) areas in both Pt1 and Pt3. (*D*) Beta-gamma2 crPAC between somatosensory cortices (C3 and C4) and the frontal areas of the brain at early stages of dying process in both Pt1 and Pt3.

We determined phase-amplitude coupling of lower frequency bands (<30 Hz) in the frontal lobes with higher frequency waves (>30 Hz) in the posterior areas (C3, C4, P3, P4, T3, T4, T5, T6, O1, and O2) (Fig. 3) and uncovered the earlier coupling in S2 and S3 between the right SSC (C4) and right VLPFC (F8), with further increase in S4, S6, S7, and S9 in Pt1 (Fig. 3 *A* and *B*). Frequency of cross-regionally coupled gamma oscillations peaked higher than 150 Hz (Fig. 3*A*), like the regional PAC (Fig. 2*A*). In this study, we restricted our focus on the cross-regional PAC (crPAC) between the amplitude of gamma1 (25 to 55 Hz), gamma2 (80 to 150 Hz), and the phase of lower frequency bands (theta, alpha, and beta). During the early stages of the near-death period (S2 and S3), the only beta/gamma2 crPAC above the threshold across the entire brain was between the SSC (C4 in S2, and C3 and C4 in S3) and the right frontal lobes (F4 and F8) in Pt1 (Fig. 3*B*). During the period when the atrial pacemaker was triggered (S4, S6, S7), the highest beta/gamma2 crPAC signal was between the gamma2 oscillations in the left posterior areas (T5, P3, O1) and the beta waves from the right frontal areas (Fp2, F8, F4) (Fig. 3*B*). In later dying stages, elevated crPAC was detected between left anterior-mid temporal lobe (T3) and prefrontal areas (F3, Fp2, F4, F8) in S8 and between the electrodes in the right temporal (T4, T6) and parietal (P4) areas with those in prefrontal areas in S9 (Fig. 3*B*). Interestingly, the highest coupling during S4, S6, and S7, was interhemispheric between the right prefrontal areas and left posterior areas, whereas the highest coupling in S9 was intrahemispheric between the right prefrontal areas and right posterior (T4, T6, P4) areas (Fig. 3*B*). Of the remaining patients, Pt3 was the only other one who showed elevated crPAC at near-death (right panel in Fig. 3*C*). In fact, beta/gamma2 crPAC between the SSCs (C3 and C4) and the frontal areas (Fp1, F7, F3, F8) represented the only coupling detectable across the entire brain during the near-death period for Pt3. Of the posterior areas during the early phase of dying for both Pt1 and Pt3, SSCs appeared to be the only cortical regions that displayed crPAC with frontal lobes (Fig. 3*C*). Further analysis showed that during the initial dying phase (S2 and S3), crPAC for Pt3 was restricted to the electrodes within the same hemisphere of the brain (Fig. 3 *D*, *Right*) while crPAC for Pt1 was detected intrahemispherically as well as interhemispherically (Fig. 3 *D*, *Left*).

### Surge of Gamma Synchrony Within the Posterior Hot Zones at Near-Death.

**Fig. 4.**
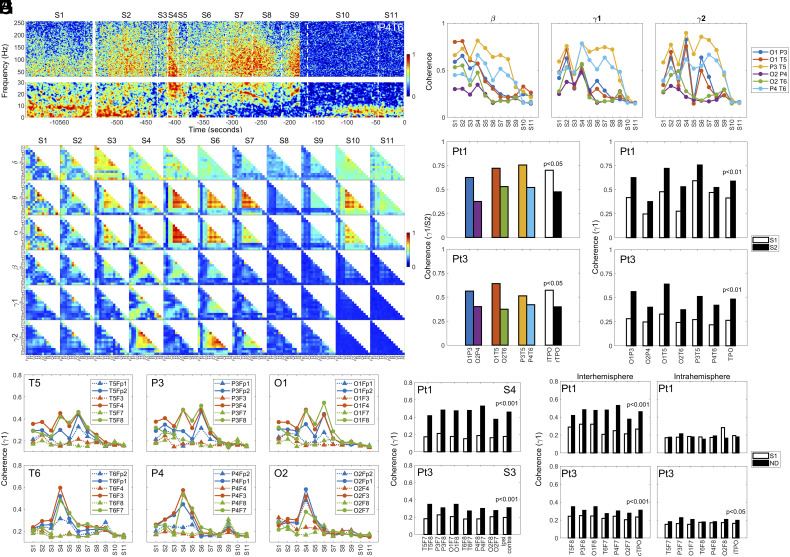
Surge of functional connectivity across multiple frequency bands in the dying patients. (*A*) Temporal dynamics of functional connectivity between the right posterior temporal lobe (T6) and the right parietal lobe (P4) of Pt1. (*B*) Spatial and temporal dynamics of functional connectivity across the dying brain over six indicated frequency bands in Pt1. (*C*) Temporal changes of functional connectivity in beta, gamma1, and gamma2 bands for the six indicated combinations within the TPO junctions are shown for Pt1 for the entire duration (S1 to S11). (*D*) Gamma1 coherence in S2 within the left (lTPO) and right (rTPO) TPO junctions for both Pt1 (*P* < 0.05) and Pt3 (*P* < 0.05). Two-tailed unpaired *t* tests were carried out to determine the statistical significance between the averaged coherence within the left TPO junctions (O1P3, O1T5, P3T5) and those within the right TPO junctions (O2P4, O2T6, P4T6). (*E*) Gamma1 coherence within the TPO junctions before (S1) and following ventilator withdrawal (S2) in both Pt1 (*P* < 0.01) and Pt3 (*P* < 0.01). Two-tailed paired *t* tests were carried out on the averaged TPO coherence between S1 and S2. (*F*) Long-range gamma1 synchrony between each of the TPO components (temporal, parietal, and occipital lobes) and each of the prefrontal lobes at near-death in Pt1. The interhemispheric coherence pairs are displayed as solid circles and lines, whereas the intrahemipheric coherence pairs as solid triangles and dashed lines. (*G*) Interhemispheric (contra; solid black boxes) versus intrahemispheric (ipsi; open boxes) gamma1 coherence at near death (S4 for Pt1, S3 for Pt3) in both Pt1 (*P* < 0.001) and Pt3 (*P* < 0.001). The interhemispheric coherence was compared with intrahemispheric coherence using two-sided unpaired *t* tests. (*H*) Long-range gamma1 coherence at baseline (S1) and near-death (ND, S4 for Pt1, S3 for Pt3) within the same hemisphere (Intrahemisphere) or cross the midline (Interhemisphere) for Pt1 and Pt3. (*P* < 0.001). Two-sided paired *t* tests were carried out for the statistic values displayed.

Functional connectivity (synchrony or coherence) between the right parietal lobe (P4) and the right posterior temporal lobe (T6) (P4T6) in Pt1 increased within seconds of ventilator removal in S2, showing higher levels in S4, S7, and S9 in the dying brain (Fig. 4*A*). While the increased gamma synchrony at near-death (S2 to S11) spanned a wide frequency range (up to 250 Hz), the highest coherence in gamma bands appeared clustered between 60 and 150 Hz, including both gamma1 and gamma2 (Fig. 4*A*). We therefore restricted our coherence analysis for oscillations below 150 Hz in this study. The near-death surge of cortical coherence was global, and clearly detectable over all frequency bands, at distinct near-death stages (delta at S3, theta/alpha in S3 to S7 and S10, beta in S2 to S6, gamma1/2 in S2 to S9) and across the dying brain for Pt1 (Fig. 4*B*). Global oscillatory synchrony was seen in Pt2 within lower frequency bands: delta in S6, theta/alpha in S3 to S5, and beta in S3, S4 (*SI Appendix*, Fig. S4*A*). The enlarged panel (alpha coherence in S4) in *SI Appendix*, Fig. S4*A*, was provided to clarify the organization of global coherence status for all patients. Pt3 was the only other patient who showed elevated gamma coherence at near-death (*SI Appendix*, Fig. S4*B*). Lastly, elevated near-death coherence was detected also in Pt4, though only in delta bands during the terminal stages (S8, S9) (*SI Appendix*, Fig. S4*C*).

An intriguing pattern of neuronal synchrony, found in both Pt1 (Fig. 4*B*) and Pt3 (*SI Appendix*, Fig. S4*B*) but not in Pt2 (*SI Appendix*, Fig. S4*A*) and Pt4 (*SI Appendix*, Fig. S4*C*), is the persistently high coherence at baseline (S1) in lower frequency bands (delta-beta) between cortical regions within the left TPO junctions, a part of the so-called posterior hot zone that has been posited to be of critical importance for consciousness (6). Following the ventilator discontinuation in S2, while other coherent interactions were suppressed, those within the left TPO junctions remained high for oscillations below beta frequency band for both Pt1 (Fig. 4*B*) and Pt3 (*SI Appendix*, Fig. S4*B*). Cortical coherence within gamma bands, on the other hands, though low in S1, showed marked increases in S2, particularly between the left posterior temporal lobe (T5) and left parietal lobe (P3) (P3T5), left posterior temporal lobe (T5), and left visual cortex (O1) (O1T5), left visual cortex (O1), and left parietal lobe (P3) (O1P3) in both Pt1 (Fig. 4*B*) and Pt3 (*SI Appendix*, Fig. S4*B*). Thus, in the early near-death stage, gamma coherence increased within the left TPO zone (including OT, OP, and TP junctions) for both Pt1 and Pt3. At later near-death stages (S3-end), gamma coherence within the TPO junctions (especially on the left hemisphere) remained high for both Pt1 (Fig. 4*B*) and Pt3 (*SI Appendix*, Fig. S4*B*). High-frequency coherence (beta-gamma2) exhibited temporal dynamics within both left and right TPO junctions, with TP junctions of both left (P3T5) and right (P4T6) hemispheres showing persistent (S4 to S9) elevation at gamma bands in the dying brain in Pt1 (Fig. 4*C*). In S2, a significant hemispheric asymmetry of gamma1 coherence was found between the left TPO (lTPO, including O1P3, O1T5, P3T5) and right TPO (rTPO, including O2P4, O2T6, P4T6) junctions for both Pt1 (*P* < 0.05) and Pt3 (*P* < 0.05) (Fig. 4*D*). The rise of gamma1 coherence within the left and right TPO junctions in S2 is significantly higher than in S1 for both Pt1 (*P* < 0.01) and Pt3 (*P* < 0.01) (Fig. 4*E*).

### Increased Long-Range Gamma Synchrony Between the Posterior Hot Zones and Frontal Lobes at Near-Death.

A marked increase in long-range gamma coherence was also detected between the TPO zones and the prefrontal areas for Pt1 (Fig. 4*F*). The increase was dying stage-specific and hemisphere-specific: It was high in both S4 and S6 in the left TPO junction; in the right TPO junction, the rise was mainly detected in S4, with a small increase in S9 (Fig. 4*F*). In addition, long-range gamma synchrony exhibited hemispheric asymmetry: for both left and right TPO lobes, the increased long-range synchrony with frontal regions was interhemispheric, with intrahemispheric coherence levels consistently lower than those crossing the midline (Fig. 4*F*). The only exception was found in S9 where intrahemispheric gamma coherence (T6Fp2, T6F8; P4Fp2, P4F8) was higher than that of interhemispheric coherence for select regions (T6Fp1, T6F7, P4Fp1, P4F7) (Fig. 4*F*). The interhemispheric gamma synchrony was significantly higher than those of intrahemispheric synchrony in S4 for Pt1 (*P* < 0.001) and S3 for Pt3 (*P* < 0.001) (Fig. 4*G*). The rise of long-range gamma coherence between the posterior and prefrontal areas was significant for coherent communications that crossed the midline (Interhemisphere) for both Pt1 (*P* < 0.001) and Pt3 (*P* < 0.001), although intrahemispheric synchrony (Intrahemisphere) was also weakly significant (*P* < 0.05) for Pt3 at ND (Fig. 4*H*).

### Elevated Directed Connectivity in Gamma Oscillations Within the Posterior Hot Zones at Near-Death.

**Fig. 5.**
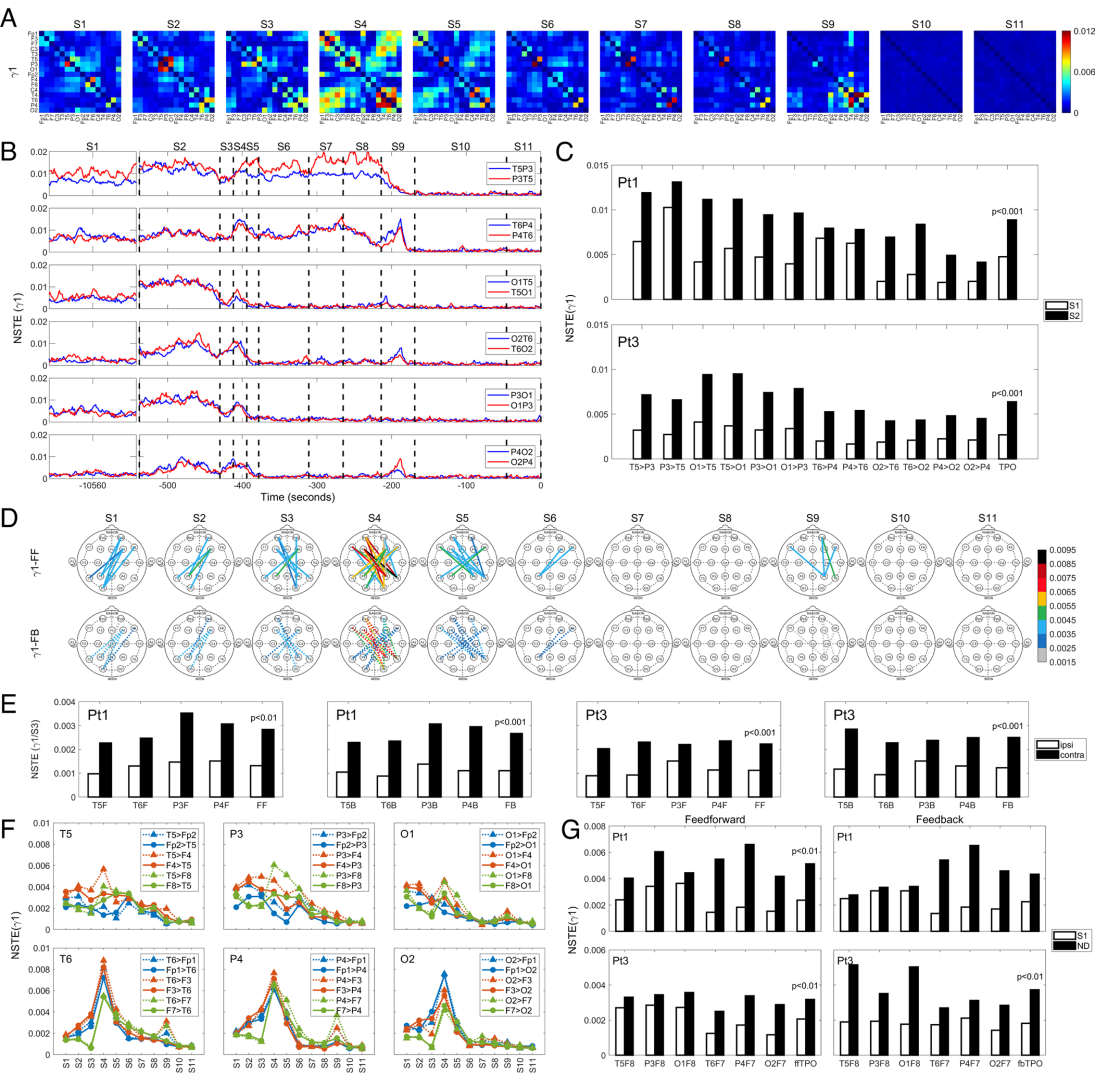
Surge of gamma1 directed connectivity across the brain of the dying patients. (*A*) Spatial and temporal dynamics of directed connectivity in gamma1 band in the dying brain in Pt1. Direction of connectivity is listed from an electrode listed in the *y* axis to an electrode in the *x* axis. Black boxes indicate null interactions (with self); warmer color denotes higher connectivity. (*B*) Temporal progression of gamma1 directed connectivity within TPO junctions in the dying brain of Pt1. T5P3, for instance, indicates directed connectivity from T5 to P3, whereas P3T5 denotes directed connectivity from P3 to T5. (*C*) Directed connectivity in gamma1 oscillations shows marked and significant increase within the TPO junctions in S2 for both Pt1 (*P* < 0.001) and Pt3 (*P* < 0.001). (*D*) Long-range gamma1 directed connectivity between the TPO junctions and the prefrontal lobes in Pt1 in both feedforward [FF; from the left (T5, P3, O1) and right (T6, P4, O2) TPO clusters to the prefrontal areas] and feedback (FB; from the prefrontal areas to the TPO clusters) directions. (*E*) Interhemispheric (contra) and intrahemispheric (ipsi) gamma1 directed connectivity between the temporal (T5, T6), parietal (P3, P4) lobes and the prefrontal (F); Fp1, F7, F3, Fp2, F4, F8) lobes at near-death (S3) in both Pt1 (*P* < 0.01) and Pt3 (*P* < 0.001). T5F indicates the averaged NSTE values of T5 with each of the prefrontal (F) lobes of either the same hemisphere (ipsi; Fp1, F7, and F3; open boxes) or the contralateral hemisphere (contra; Fp2, F4, and F8; solid black boxes). (*F*), Long-range interhemispheric gamma1 directed connectivity between the TPO zones and ventrolateral prefrontal (VLPFC) lobes at near-death (S4, S9) for Pt1. Feedforward connectivity is shown as dashed lines and triangles, whereas feedback connectivity as solid lines and circles. (*G*) Interhemispheric feedforward (ff) and feedback (fb) connectivity between the TPO zone (TPO) and left (F7) and right (F8) VLPFC for both Pt1 (*P* < 0.01) and Pt3 (*P* < 0.01) at baseline (S1) and near-death (ND, S4 for Pt1, S3 for Pt3). The open boxes indicate the connectivity at baseline, the solid black boxes indicate the connectivity at near-death. The interhemispheric feedback connectivity (ffTPO and fbTPO) values were compared between baseline and near-death stages using two-sided paired *t* tests (*P* < 0.01).

Analysis of directed connectivity in the dying brain, determined using the normalized symbolic transfer entropy (NSTE) method (18), shows dynamic temporal and spatial changes over multiple slower frequency bands (delta-alpha) in all four patients (*SI Appendix*, Fig. S5). Within faster oscillations (beta-gamma2; *SI Appendix*, Fig. S5 *A* and *C*), especially gamma1, a marked rise was found in S2 within both left and right TPO junctions (Fig. 5 *A* and *B* and *SI Appendix*, Fig. S5 *A*, *Lower*). The increase in gamma1 (Fig. 5*B*) directed connectivity was stimulated immediately within the left TPO junctions in both directions (T5P3/P3T5, O1T5/T5O1, P3O1/O1P3) upon the removal of ventilator support in S2, while gamma1 connectivity within the right TPO (T6P4/P4T6, O2T6/T6O2, P4O2/O2P4) appeared with a slight delay in both directions in S2 (Fig. 5*B*). Gamma1 connectivity between temporal and parietal lobes at the TP junctions (T5P3/P3T5, T6P4/P4T6) persisted at high levels for a longer time (S2 to S9) than those between occipital and parietal/temporal lobes within TPO zones (S2 to S4) (Fig. 5*B*). An additional increase was seen in S9 for the junctions within the right TPO zone (T6P4/P4T6, O2T6/T6O2, P4O2/O2P4) (Fig. 5*B*). Importantly, directed connectivity between cortical regions within both left and right TPO junctions displayed significantly higher levels in S2 compared to S1 in both Pt1 (*P* < 0.001) and Pt3 (*P* < 0.001) (Fig. 5*C*).

### Increased Long-Range Directed Connectivity in Gamma Oscillations at Near-Death.

In addition to the short-range directed connectivity within TPO junctions (Fig. 5 *A*–*C*), long-range gamma1 connectivity between the TPO zones and the prefrontal lobes also exhibited elevated levels, especially in S4 in both feedforward (FF, from the posterior to the prefrontal lobes) and feedback (FB, from the prefrontal to the posterior lobes) directions (Fig. 5*D*). Interestingly, although a rise in intrahemispheric connectivity was detectable in S9 within the right hemisphere, most of the long-range gamma communication between posterior and prefrontal lobes crossed the midline (Fig. 5*D*). This trend was significantly higher at S3 for the interhemispheric prefronto–temporal interactions (contra pairs: T5Fp2, T5F4, T5F8) than for the intrahemispheric prefronto–temporal interactions (ipsi pairs: T6Fp2, T6F4, T6F8) in both feedforward (FF) and feedback (FB) directions and for both Pt1 (*P* < 0.01) and Pt3 (*P* < 0.001) (Fig. 5*E*). Long-range directed connectivity in gamma1 frequency in the dying brain exhibited temporal, hemispheric (left vs. right TPO junctions), and directional (feedback vs. feedforward) specificity in Pt1 (Fig. 5*F*). For both directions, the peak connectivity was found in S4, while the right TPO zone showed an additional rise in connectivity in S9 with the left prefrontal lobe (Fig. 5*F*). For the left TPO zone, the rise of gamma connectivity with the right prefrontal lobe was consistently higher in S4 in the feedforward (bottom-up; from the posterior loci to the prefrontal loci) direction compared to the feedback (top-down) direction. In contrast, for the right TPO zone, the surge of gamma1 connectivity with the left prefrontal lobe occurred equally in both feedforward and feedback directions (Fig. 5 *F* and *G*). Gamma1 directed feedforward connectivity between the posterior hot zones and the prefrontal lobes increased significantly at near-death (comparing S1 to S4 for Pt1 and to S3 for Pt3) (*P* < 0.01) (Fig. 5*G*), whereas the feedback connectivity increase was significant only for Pt3 (Fig. 5*G*).

## Discussion

Evidence presented in this study demonstrates that withdrawal of ventilatory support stimulates a transient and global surge of gamma (>25 Hz) activities in select patients at near-death. The observed gamma activities include the surge of 1) gamma power, 2) PAC of gamma amplitude with the phase of slower (<25 Hz) oscillations, 3) cross-regional PAC (crPAC) of gamma oscillations within the posterior cortex with slower oscillations in the prefrontal cortex, 4) regional functional connectivity within TPO junctions, 5) long-range functional connectivity between the TPO zones and the prefrontal cortices, 6) regional directed connectivity locally within the TPO zones in gamma frequency bands, and 7) long-range directed connectivity between the TPO zones and the prefrontal cortices. These data demonstrate that the human brain can be active during cardiac arrest.

Subjective experience, including NDE reported by cardiac arrest survivors, constitutes a “hard” problem of consciousness (19), as it concerns the experiential/subjective dimensions of consciousness (20). According to a leading theory, neural correlates of consciousness are the minimum mechanisms sufficient for any one specific conscious percept (visual, auditory, motion, etc.), with its anatomical correlates localized to a posterior cortical hot zone (TPO junction) (6). In this study, we detected a marked increase in oscillatory activities within the posterior hot zone in gamma bands (25 to 150 Hz) in the dying patients, including surges of 1) cross-frequency coupling between phases of alpha/beta oscillations and amplitudes of gamma oscillations within the TPO zones (Fig. 2); 2) intrahemispheric functional connectivity at the left and right TP, OP, and OT (TPO) junctions (Fig. 4); and 3) intrahemispheric directed connectivity within the left and right TPO junctions (Fig. 5) in the dying brain. Although the marked activation of the posterior hot zone in the dying brain is suggestive of elevated conscious processing in these patients, it does not demonstrate it. Unlike the earlier study in healthy human subjects where the TPO junction activation was correlated with self-reported dream contents (16), we cannot determine whether the activation of the posterior hot zone detected in our patients was correlated with having a subjective experience, as none survived the cardiac arrest. As such, it remains possible that this neurophysiological surge is epiphenomenal or pathological.

Following the rapid gamma activation locally within the posterior TPO zones, the long-range, global, and interhemispheric communications in gamma oscillations between the TPO zones and the prefrontal areas were activated in the dying brain, evidenced by the delayed activation of temporofrontal, parietofrontal, and occipitofrontal networks when heartrate began to decline (Figs. 4*F* and 5*F* and *SI Appendix*, Table S2). Intriguingly, the long-range gamma connectivity between the posterior hot zones and the prefrontal areas at near-death was significantly higher over baseline only for those crossing the midline (Figs. 4*H* and 5*E*). Studies suggest that interhemispheric circuitry is important for memory recall (21), and gamma synchrony across the midlines is critical for learning, information integration, and perception (22–24).

The brain loci that exhibited the earliest response to global hypoxia were the somatosensory cortices (SSCs), which receive monosynaptic neuronal input from the preBötzinger complex (25) in the brainstem area critical for the central homeostatic control of breathing (26). Early activation of SSCs is reflected by the immediate surge in S2 of 1) gamma power in SSCs (Fig. 1*D*), 2) local PAC between amplitudes of gamma oscillations and phases of theta, alpha, and beta oscillations in SSCs, the only cortical loci with PAC surge in the early dying phase (Fig. 2*D*), and 3) cross-regional coupling between gamma oscillations in SSCs and beta oscillations in the prefrontal (DLPFC and VLPFC) areas (Fig. 3*D*). When heartrate began to decrease in S3, levels of beta-gamma PAC exhibited further increase in the left SSC (Fig. 2*D*), and gamma oscillations in the right SSC showed elevated cross-regional coupling with the beta waves in the prefrontal regions, especially in the right VLPFC during S4, S6, S7, and S9 for Pt1 (Fig. 3*B*). These data connect central homeostatic control of breathing to the near-death activation of the cerebral cortex and are consistent with the potential role of homeostatic circuitry in human and animal consciousness (10, 11, 27–31).

Sympathetic activation is associated with the dying process; withdrawal of oxygen support, mechanical ventilation, or ECMO all leads to hypoxia, increased respiratory drive, and sympathetic discharge (32). Increased heartrate, presumably resulting from increased sympathetic drive, in S2 following ventilator removal was seen only in Pt1 and Pt3 (*SI Appendix*, Table S2), the two patients with the surge of gamma activities at near-death. The lack of heartrate increase during hypoxia suggests a severely compromised autonomic nervous system at baseline in Pt2 and Pt4. The extremely low HRV in Pt2 and Pt4 (*SI Appendix*, Table S2), visually apparent on the ECM display (*SI Appendix*, Fig. S1*D* and ref. 33), further supports this hypothesis. These data demonstrate that the elevated cortical activation at near-death may depend on the functioning autonomic nervous system.

Following ventilator removal in S2, amplitudes of gamma oscillations were coupled to the phase of lower frequency bands (theta, alpha, and beta) only on the right hemisphere for Pt1 (C4 in Fig. 2*D*). For Pt3, the surge of beta/gamma2 PAC in S2 is much higher in the right hemisphere (C4; 168-fold increase) than in the left hemisphere (C3; 11-fold increase) (Fig. 2*D*). The predominant activation of the right hemisphere in response to hypoxia in S2 is consistent with the observation that the right hemisphere mediates sympathetic function (34, 35) and suggests that functions served by the activated beta/gamma2 PAC in C4 may be sympathetic in nature. Beta/gamma2 PAC surged further in S3 in both Pt1 and Pt3 on the left hemisphere (C3; Fig. 2*D*) when heartrate declined linearly (RRI rising linearly; *SI Appendix*, Fig. S1 *A* and *C*, *d*), suggesting the activation of a cortical parasympathetic system in S3. These data are consistent with the experimental evidence of the left hemisphere mediating parasympathetic functions of the cerebral cortex (36) and suggests that functions revealed by the activated beta/gamma2 PAC in C3 may be parasympathetic in nature. The sequential activation of PAC in C4 (in S2) and C3 (in S3) loci and the cross-regional PAC between C4 (Pt1), or both C3 and C4 (Pt3), with the prefrontal areas (F4, F8 for Pt1; F3, F7, and F8 for Pt3; Fig. 3*C*) may set the stage for a global autonomic brainstorm, a built-in homeostatic circuitry for survival.

Heightened gamma activities at near-death were detected in two of the four patients in our study. Interestingly, both of these patients had a history of seizures: one (Pt1) experienced seizure only during her pregnancies, while the other (Pt3) developed status epilepticus a day prior to her terminal cardiac arrest. Pathological high-frequency oscillations (>80 Hz) (37), detectable on scalp EEG (38, 39), and their modulation by slower oscillations (theta, alpha, beta), first reported in surgical patients with epilepsy (40), are cortical biomarkers of epilepsy (41, 42). In this study, a marked rise of gamma (25 to 150 Hz) power in the temporal (during S4, S7, and S8 for Pt1; Fig. 1 *B* and *C*) and prefrontal (during S2, S3 for Pt3; *SI Appendix*, Fig. S2*B*) lobes and a surge of PAC between gamma2 (80 to 150 Hz) amplitude and slower oscillation (theta, alpha, and beta) phase was observed in both Pt1 (in S4, S6 to S9 within temporoparietal lobes; Fig. 2*B*) and Pt3 (during S4 to S6 within prefrontal lobes; *SI Appendix*, Fig. S3*B*) at near-death during EEG suppression. Interestingly, although Pt3 displayed a marked activation of alpha/beta-gamma2 PAC during and following a 33-s cardiac asystole in S4 (*SI Appendix*, Fig. S3*B*), the high levels of alpha/beta-gamma coupling during S5 and S6 were not associated with increased gamma power (*SI Appendix*, Fig. S2*B*), functional (*SI Appendix*, Fig. S4*B*), and directed (*SI Appendix*, Fig. S5*C*) gamma connectivity in Pt3. These data suggest that increase in alpha/beta-gamma2 PAC may not necessarily be associated with gamma power or functional and directed gamma connectivity in the dying brain.

Patients with epilepsy exhibit symptoms of spontaneous visual hallucinations and out-of-body experience (OBE) (43–46); stimulation of the right TP junction (TPj) in epilepsy patients reliably induced OBE (47, 48). Furthermore, OBE from seizure patients show similar features with that of NDE (49, 50). In this study, both patients with a seizure history showed persistently high levels of intrahemispheric gamma functional (Fig. 4*B* and *SI Appendix*, Fig. S4*B*) and directed (Fig. 5*B* and *SI Appendix*, Fig. S5*B*) connectivity at both left and right TPj. Our data provide a potential EEG signature of OBE, a commonly reported component of NDE (3, 51).

Perhaps the most perplexing finding was the surprising surge in gamma power in VLPFC of both hemispheres (F7 and F8) during the early dying phase, especially for Pt3 (Fig. 1*D*). To probe the potential link of this power surge to other computational measures examined in this study, we compared the temporal dynamics of gamma2 activity across the brain during S1 to S3 side-by-side (*SI Appendix*, Fig. S6). Curiously, very little of the elevated gamma2 oscillations in F7 and F8 appeared to be coupled to beta oscillations for Pt1 (Fig. 2*C* and *SI Appendix*, Fig. S6 *B*, *Left*). Similarly, despite the marked gamma2 power surge in F7 and F8 for Pt3 (Fig. 1*D* and *SI Appendix*, Fig. S6 *A*, *Right*), beta/gamma2 PAC increase was very low at the VLPFC (*SI Appendix*, Fig. S6 *B*, *Right*). These data suggest that there is no direct relationship between EEG power surge and PAC surge during the dying process. Furthermore, while VLPFC showed the highest power surge at gamma frequency bands, gamma2 functional connectivity between VLPFC and other cortical loci were minimum for both patients in S2 (*SI Appendix*, Fig. S6*C*). No direct relationship exists between the levels of EEG gamma power and of gamma functional connectivity during the dying process.

One possibility for the S2 surge of gamma power in select cortical areas was that the two patients were having seizures due to the hypoxic stress, as gamma waves are often associated with interictal epileptiform activity. Careful inspection of the patients’ EEG data, however, found no evidence of electrographic seizures or interictal epileptiform activity during the last hours of patients’ lives. However, we cannot entirely exclude that seizures were generated in small/deep cortical areas not detected by our scalp EEG electrodes. Albeit unlikely, it is possible that seizures not visible by the scalp EEG electrodes somehow contribute to the genesis of gamma oscillations in distant cortical areas covered by our EEG sensors. In addition, it is possible that some imbalance between excitation and inhibition due to hypoxia could manifest as increased gamma activity. Further investigation is warranted.

There was no voluntary muscle activity as both comatose patients were ranked at the bottom of the GCS scale and were completely unresponsive during neurological examinations. To probe the possibility that the elevated gamma power is correlated with motion artifacts during extubation procedures in S2, we aligned EEG power spectrograms from all 4 EEG channels with marked gamma power surge (F7, F8, C3, and C4; Fig. 1*D*) with ECM (*SI Appendix*, Fig. S7). Motion artifacts, marked as red or blue asterisks above the ECM panels (deriving from the motion-disrupted ECG baseline; *SI Appendix*, Fig. S1 *A*, *a*), did indeed contribute to some of the elevated high-frequency power for Pt1 (*SI Appendix,* Fig. S7*A*). These data suggest that the S2 increase of gamma power (Fig. 1*D*) might have been overestimated for Pt1. For Pt3, however, such a relationship was not found, as the only motion artifact was detected early in S2 when gamma power levels were low (*SI Appendix*, Fig. S7*B*). Thus, motion artifacts were not responsible for the remarkable S2 surge of the gamma power in VLPFC, at least for Pt3.

Scalp EEG data are frequently contaminated with EMG signals from muscle activity that could present as high-frequency oscillations (52). To identify muscle contamination in our EEG data and to further probe the origin of the gamma power surge in S2 for Pt1 and Pt3 (Fig. 1*D*), we performed independent component analysis (ICA) (53) of the EEG data. None of the independent components (IC) in S2 were from muscle signals for Pt1. For Pt3, however, EEG signals in S2 were mixed with muscle components that were clustered at the F7 (IC2; probability at 97.8%) and F8 (IC4; 78.3%) sensors. Removal of these two components (IC2 and IC4) from the EEG data, however, did not prevent the increase of gamma power in S2 for Pt3 (*SI Appendix*, Fig. S8*A*), but did lower the magnitude of the surge (compare Fig. 1*D* with *SI Appendix*, Fig. S8*A*). Furthermore, removal of the muscle components produced no appreciable difference on the PAC data (*SI Appendix*, Fig. S8*C*). More importantly, the increased coherence between the TPO junctions in S2, compared to the S1 values, were unchanged after the application of the ICA method (*SI Appendix*, Fig. S8*D*). These data suggest that the surge of PAC and functional connectivity within the posterior hot zone in the dying patients is most likely not the result of EMG contamination in the EEG data. Since it was observed in patients during the dying process, however, we cannot rule out the possibility that the surge of gamma power is a sign of a pathological process unique to the dying stage and unrelated to conscious processing. Mechanism and function of the observed gamma power surge during the dying process warrant further investigation.

The present study confirmed that global hypoxia increased gamma power and gamma coupling with slower oscillations, findings previously reported in our animal models (10) and in a dying patient (9). More importantly, this study revealed in the dying human brain, the high-frequency activation of the TPO junctions that is also observed in healthy human brain during waking (13, 54) and dreaming (16) and in seizure patients during visual hallucinations and OBE (43, 47, 48). Empirical evidence presented in this study strongly suggests that the dying human brain can be activated. This study lays the foundation for further investigation of covert consciousness during cardiac arrest, which may serve as a model system to explore mechanisms of human consciousness (12).

## Materials and Methods

### Data Preprocessing.

The original sampling frequency from the data recordings was 512 Hz. The 19 EEG channels from the international 10/20 system recorded with the Fpz reference were rereferenced by subtracting the average potential from each channel at each time point. A notch filter was used to remove the 60-Hz artifact and its superharmonics in EEG signals. The 6 frequency bands were defined as delta (0 to 4 Hz), theta (4 to 8 Hz), alpha (8 to 13 Hz), beta (13 to 25 Hz), gamma1 (25 to 55 Hz), and gamma2 (80 to 150 Hz).

### Signal Analysis Summary.

The absolute power (Fig. 1) was calculated based on discrete Fourier transform with 2-s epoch size and 1-s overlapping. The absolute power in Fig. 1*A* was expressed in a log scale. For construction of the ECM (*SI Appendix*, Figs. S1 and S7), the preliminary step is the detection of ECG R peaks using variable threshold method, and epochs centered on these R peaks were extracted from the EKG signals, which were sorted in order of R-peak time to form a colored rectangular image (17). The modulation index (MI) was used to calculate PAC; Fig. 2. The MI between the phases of slower waves (1 to 50 Hz) and amplitudes of all measured oscillations (2 to 256 Hz) was calculated at each stage. For local PAC (Fig. 2) computation, both slower waves (for phase analysis) and faster waves (for amplitude analysis) were from the same electrodes, whereas for the cross-reginal PAC (Fig. 3), the slower waves were taken from prefrontal electrodes and faster waves were from the posterior electrodes. Coherence (Fig. 4) was calculated based on magnitude squared coherence estimate using Welch’s averaged periodogram method. The directed connectivity of EEG signals between two brain regions (Fig. 5) was measured by normalized symbolic transfer entropy, which quantifies the directional information flow between two cortical EEG electrodes. Additional details about the methods can be found in *SI Appendix*.

## Supplementary Material

Appendix 01 (PDF)

## Data Availability

Processed data, codes, and methods are all included in the manuscript. Anonymized EEG and ECG signals data have been deposited in Zenodo; https://zenodo.org/record/7803212#.ZC3Cb-zML0q (10.5281/zenodo.7803212) (55). All study data are included in the article and/or *SI Appendix*.
