# Supplementary material for: Surge of neurophysiological coupling and connectivity of gamma oscillations in the dying human brain
Source: Proc Natl Acad Sci U S A. 2023 May 1;120(19):e2216268120. doi: 10.1073/pnas.2216268120 (PMC10175832; doi:10.1073/pnas.2216268120)
Supplement: Supplementary file 1 — Appendix 01 (PDF) [file pnas.2216268120.sapp.pdf]

Supporting Information for

Surge of neurophysiological coupling and connectivity of gamma oscillations in the dying human brain

By Gang Xu, Temenuzhka Mihaylova, Duan Li, Fangyun Tian, Peter M. Farrehi, Jack M. Parent, George A. Mashour, Michael M. Wang, Jimo Borjigin*

*Email: borjigin@umich.edu

This PDF file includes:

SI Materials and Methods

Tables S1, S2

Figures S1 to S8

SI Materials and Methods

Analysis of Power Spectrum. The spectrogram in Fig. 1a was calculated based on discrete Fourier transform with 2-s epoch size and 1-s overlapping for each frequency bin (0.5–256 Hz with 0.5 Hz bin size; spectrogram.m in MATLAB Signal Processing Toolbox; MathWorks Inc.). Each epoch is windowed with a Hamming window. The absolute power was expressed in log scale (Fig. 1a). The mean of absolute powers was calculated to render topographic maps for all baseline/near-death stages and 6 frequency bands (Fig. 1b and *SI Appendix,* Fig. S2a-c). The topographic map of 19 EEG channels (Fig. 1b and *SI Appendix,* Fig. S2a-c) was rendered by MATLAB function topoplot.m from EEGLAB Toolbox version 10.2.5.5a.

Analysis of Cross-Frequency Coupling. Modulation index (MI) was used for measuring the phase-amplitude coupling (PAC) between gamma waves and lower frequency bands. To measure MI for a given EEG data, EEG signals were first filtered via convolution with a complex Morlet wavelet with width of seven (central frequencies: 1–50 Hz for phases and 2–256 Hz for amplitude envelopes with 2-Hz bin). For each filtered signal, the instantaneous phases $\phi_{LF}(t)$ of a lower-frequency band taken by applying Hilbert transform and the amplitude envelope $A(t)_{HF}$ of a higher-frequency band of EEG signal were combined into one composite, complex-valued signal $Z(t) = A(t)_{HF} \cdot e^{-i\phi_{LF}(t)}$. If the probability density function of $Z(t) = real[Z(t)] + imag[Z(t)]$ in the complex plan is not radially symmetric, it means that $A(t)_{HF}$ and $\phi_{LF}(t)$ share mutual information, or that the distribution of $\phi_{LF}(t)$ is nonuniform. MI is defined as the absolute value of the average of $Z(t)$ and measures the degree of asymmetry of the probability density function. MI is used as a metric of coupling between two frequency bands (phases of a lower-frequency band and amplitudes of a higher-frequency band). To test the significance of the asymmetry, 50 surrogate data were generated by shuffling the high-frequency amplitude envelope $A(t)_{HF}$ after dividing it into 1-s sections. This surrogate data retains the mean, variance, and power spectrum of the original signal while removing the temporal ordering between amplitudes. MI was calculated with the population of 50 shuffled amplitude envelopes and phases of a low-frequency signal, generating 50 MI values. A MI value was deemed significant if it reached the top 5% of this distribution of surrogate data. For each epoch, MI of original data and 50 MI of shuffled data were calculated over all pairs of frequency bands. When MI values of certain frequencies were significant, we used the MI values for the average, otherwise, padded with zero. An example of PAC for channel T4 was shown with

lower-frequency 0-50 Hz and higher-frequency 30-250 Hz (Fig. 2a). For each baseline/near-death stage, different types of PAC were investigated between the four slow-frequency bands [delta (1–4 Hz), theta (4–8 Hz), alpha (8–13 Hz), and beta (13-25 Hz)] and two fast-frequency bands [gamma1 (25–55 Hz) and gamma2 (80–150 Hz)] (Fig. 2b). The topographic map of PAC of 6 types (delta-gamma1, theta-gamma1, alpha-gamma1, alpha-gamma1, theta-gamma2, alpha-gamma2, and beta-gamma2) over the 19 EEG channels (Fig. 2b and *SI Appendix,* Fig. S3a-c) was rendered by MATLAB function topoplot.m from EEGLAB Toolbox version 10.2.5.5a.

Analysis of Cross Regional PAC. The same MI assessment was applied to the cross-regional PAC (crPAC) between low-frequency from the prefrontal region and high-frequency from the posterior cortical regions. A representative crPAC in Pt1 (Fig. 3a) was calculated between the phase of lower frequency (1-50 Hz) oscillations in F8 and the amplitude of high frequency (30-256 Hz) in C4. The spatial and temporal distribution of crPAC between the phase of beta oscillations in the prefrontal lobes (Fp1, F7, F3, Fp2, F4, F8) and the amplitude of gamma2 oscillations in the posterior areas (C3, C4, T3-T6, O1, and O2) at baseline (S1) and near-death (S2-S11) was displayed topographically using lines of different colors with a threshold of 0.04 and an incremental step of 0.02 for Pt1 (Fig. 3b).

Analysis of Coherence. The coherence between EEG channels was measured by amplitude squared coherence $C_{xy}(f)$ (mscohere.m in MATLAB signal toolbox; MathWorks, Inc.), which is a coherence estimate of the input signals *x* and *y* using Welch's averaged, modified periodogram method. The magnitude squared coherence estimate $C_{xy}(f)$ is a function of frequency with values between 0 and 1 that indicates how well *x* corresponds to *y* at each frequency.

$$C_{xy}(f) = \frac{|P_{xy}(f)|^2}{P_{xx}(f)P_{yy}(f)}, 0 \leq C_{xy}(f) \leq 1, \quad [1]$$

where $P_{xx}(f)$ and $P_{yy}(f)$ are the power spectral density of x and y and $P_{xy}(f)$ is the cross-power spectrum spectral density. The magnitude squared coherence estimate was calculated at each epoch and frequency bin (over the frequency range of 0.5–256 Hz) for each baseline/near-death stage. Specifically, EEG signal was segmented into 2-s epochs with 1-s overlapping (Fig. 4a upper panel), and 4-s epochs with 2-s overlapping

(Fig. 4a lower panel). For each baseline/near-death stage, the mean coherence among all 120 pairs of 16 EEG channels was calculated with 2-s epochs and 1-s overlapping to form triangle grid plots (Fig. 4b and *SI Appendix,* Fig. S4a-c). The electrodes indicated on the left side of each of triangular matrices (from the top to the bottom of the Y-axis) are: F3, F7, C3, T3, P3, O1; Fp2, F4, F8, C4, T4, T6, P4, and O2, while those indicated on the X-axis of each of the matrices (from the left to right) are: Fp1, F3, F7, C3, T5, P3, O1; Fp2, F4, F8, C4, T4, T6, and P4.

Analysis of Directed Connectivity. The directed connectivity between EEG channels was measured by normalized symbolic transfer entropy (NSTE), which is a nonlinear and model-free estimation of directed functional connection based on information theory. STE measures the amount of information provided by the additional knowledge from the past of the source signal $X(X^P)$ in the model describing the information between the past $Y(Y^P)$ and the future $Y(Y^F)$ of the target signal Y, which is defined as follows:

$$STE_{X\to Y} = I(Y^F; X^P|Y^P) = H(Y^F|Y^P) - H(Y^F|X^P, Y^P), \qquad [2]$$

where $H(Y^F|Y^P)$ is the entropy of the process Y^F conditional on its past. Each vector for Y^F, X^P, and Y^P is a symbolized vector point. For the appropriate embedding parameters of symbolized vector, three embedding parameters—embedding dimension (d_E), time delay (τ), and prediction time (δ)—are needed. Here we selected the parameter set that provides the maximum information transfer from the source signal to the target signal as the primary connectivity for a given EEG dataset. By investigating the NSTE in the broad parameter space of d_E (from 2 to 10) and τ (from 1 to 30), we fixed the embedding dimension (d_E) at 3, which is the smallest dimension providing a similar NSTE, and found the time delay (τ) producing maximum NSTE. In this parameter space, a vector point could cover from 11.7 ms (with τ = 1 and d_E = 3) to 351 ms maximally (with τ = 30 and d_E= 3). By taking the maximum NSTE as the primary connectivity for a given EEG dataset, all other processes are nonparametric without subjective decisions for embedding parameters. The prediction time was determined with the time lag (from 1 to 100, 3.9–390 ms), resulting in maximum cross-correlation, assuming the time lag as the interaction delay between the source and target signals.

The potential bias of STE for a given EEG dataset was removed with a shuffled data, and the unbiased STE is normalized as follows:

$$NSTE_{X\to Y} = \frac{STE_{X\to Y} - STE_{X\to Y}^{Shuffled}}{H(Y^F|Y^P)} \in [0,1], \quad [3]$$

Where $STE_{X\to Y}^{Shuffled} = H(Y^F|Y^P) - H(Y^F|X_{Shuff}^P, Y^P)$. X_{Shuff}^P is a shuffled data by replacing the first half to the other half of data. $STE_{X\to Y}^{Shuffled}$ estimates a bias generated by characteristic of given EEG data, not by true causality. Therefore, NSTE is normalized STE (dimensionless), in which the bias of STE is subtracted from the original STE and then divided by the entropy within the target signal, $H(Y^F|Y^P)$. In EEG connectivity, NSTE represents the fraction of information in the target EEG channel not explained by its own past and explained by the source EEG channel. We first filtered EEG into six frequency bands and segmented the filtered EEG data into 10-s-long EEG epochs with 9-s overlapping. Then, feedback and feedforward were sequentially calculated at each epoch. For each connectivity pair, the NSTE data was averaged across each baseline/near-death stages for gamma1 band (Fig. 5a) and for all 6 frequency bands (*SI Appendix,* Fig. S5a-d) to differentiate the connectivity characteristics between the 16 different brain regions. The EEG channel labels on the Y-axis indicated the source of directed connectivity, while the EEG labels on the X-axis indicated the destination. The electrodes indicated on the left side of each of the matrices (from the top to the bottom of the Y-axis) are: Fp1, F3, F7, C3, T3, P3, O1; Fp2, F4, F8, C4, T4, T6, P4, and O2, while those indicated on the X-axis of each of the matrices (from the left to right) are: Fp1, F3, F7, C3, T5, P3, O1; Fp2, F4, F8, C4, T4, T6, P4 and O2. To test the difference of directed connectivity in gamma1 oscillations within the TPO junctions between S1 and S2, two-sided paired t-tests were carried out (Fig. 5c). Strength of the long-range directed connectivity in gamma1 band between each of the TPO components and each of the indicated prefrontal lobes (Fig. 5d) was indicated with colored lines with a threshold of 0.0015 and a step of 0.001. To test the directed gamma1 connectivity between the temporal (T5, T6), parietal (P3, P4) lobes and the prefrontal (F; Fp1, F7, F3, Fp2, F4, F8) lobes at S3, two-sided unpaired t-tests were carried out between interhemisphere and intrahemisphere (Fig. 5e). Quantitative long-range interhemispheric gamma1 directed connectivity between the TPO junctions and frontal lobes were shown with feedforward as dashed lines and triangles, whereas feedback connectivity as solid lines and circles (Fig. 5f). To test the interhemispheric connectivity between the TPO junctions and left (F7) and right (F8) VLPFC, two-sided paired t-tests were carried out for baseline (S1) and near-death (S4 for Pt1 and S3 for Pt3) in Fig. 5g.

Heartrate variability (HRV) analysis. Average HRV measurements were obtained for each patient during baseline (S1). Due to the presence of two premature atrial contraction (PAC) beats near the end of the S1 segment in Pt3 (*SI Appendix*, Fig. S1Cd), the first 50 sec of the S1 fragment was used for the analysis of SDNN. We measured the mean R-R interval (mean RRI) and standard deviation of all normal-to-normal intervals (SDNN).

Statistical Analyses. All statistical analyses performed using software GraphPad Prism (GraphPad Software Inc.). Paired t test was carried out between two groups of dependent samples from different states (baseline or near-death). Unpaired t test was carried out for two groups of independent samples from different locations.

SI Table S1. Demographic and clinical information of the patients

Characteristics	Patient1 (Pt1)	Patient2 (Pt2)	Patient3 (Pt3)	Patient4 (Pt4)
**Age (y)**	24	57	77	86
**Sex**	Female	Female	Female	Male
**Cardiovascular disease history**	Long QT syndrome (QT interval of 530 ms)	Heart transplant 20 years prior to her terminal visit to UM	None	Received coronary artery bypass surgery
**Other medical history**	Seizures	End stage renal disease (listed for renal transplant), hypertension, neuropathy, sleep apnea, obesity	Hypertension, hypothyroidism	None
**Causes of UM-NICU* admission**	At home cardiac arrest (down for >10min)	At home syncope (unconscious for a few minutes)	At home acute onset of nausea, vomiting, and right sided facial droop	Near drowning (under water for 5 min)
**Number of defibrillations required (ECG detected)**	3 (ventricular fibrillation, Torsade)	3 (ventricular tachycardiac, ventricular fibrillation, and Pulseless electrical activity)	None	1 (Pulseless electrical activity)
**Medical support/procedures provided at the UM-NICU**	Transvenous pacemaker, ventilation, hypothermia	ECMO#, ventilation, hypothermia	Surgical evacuation of the intraparenchymal hematoma (left parietal occipital craniotomy); ventilation	Ventilation, hypothermia
**Main findings at UM-NICU**	Nonresponsive, except intact gag reflex/Low left ventricular systolic function	Severe encephalopathy based on EEG	Intraventricular hemorrhage of the left parieto-occipital lobe/ Nonresponsive, except intact gag and cough reflex/Nonconvulsive status epilepticus	EEG burst suppression
**Glasgow coma scale (GCS)**	3	3	4	3
**Findings of the terminal CT head scan**	Cerebral herniation	No acute brain abnormality	Partial evacuation of extensive left parieto-occipital intraparenchymal hemorrhage	No acute brain abnormality

*: University of Michigan NeuroIntensive Care Unit
#: Extracorporeal membrane oxygenation

SI Table S2. ECG features in each stage of the dying process of the patients at near-death

Patients (States)	Relevant Features	Major features of ECG signals										
		**S1**	**S2**	**S3**	**S4**	**S5**	**S6**	**S7**	**S8**	**S9**	**S10**	**S11**
**Pt1 (S1-S11)**	Duration (s)	120	108	18	18	16	67	46	51	45	123	46
	Avg. RRI# (s)	0.72	0.64	0.68	0.81	0.78	0.82	1.63	7.03	4.1	1.85	3
	Avg. HR* (bpm)	83	94	88	74	77	74	37	9	15	32	20
	ECG features	Sinus rhythm, SDNN$=11	Sinus beats with baseline artifact	Sinus rhythm with declining HR	Atrial paced rhythm with normal AV^ conduction	Sinus rhythm with declining HR	Atrial paced rhythm with normal AV conduction	2:1 second degree AV block	14s asystole with irregular idioventricular rhythm	Idioventricular rhythm	Sinus rhythm with notched P-waves	Sinus rhythm with intraventricular conduction delay
**Pt2 (S1-S8)**	Duration (s)	100	100	87	157	96	216	191	773			
	Avg. RRI (s)	0.62	0.62	0.63	0.86	1.41	1.4	2.54	3.6			
	Avg. HR (bpm)	96	96	96	70	43	43	24	17			
	ECG features	Sinus rhythm with ST-elevation, SDNN=2.4	Sinus rhythm with ST-elevation	Sinus rhythm with ST-elevation	ST-elevation with declining HR	Junctional rhythms with peaked T-waves	Idioventricular rhythms	Irregular idioventricular rhythms	Agonal idioventricular rhythms			
**Pt3 (S1-S8)**	Duration (s)	100	454	76	33	32	47	208	243			
	Avg. RRI (s)	0.68	0.65	1.07	NA	1.49	1.55	1.42	1.6			
	Avg. HR (bpm)	88	93	56	NA	40	39	42	37			
	ECG features	Sinus rhythm, SDNN=4.6	Sinus rhythms	Sinus rhythm with declining HR	33s asystole	Split R-peaks with no P-waves	Junctional rhythms	Sinus bradycardia	Sinus rhythm with lengthening of P-waves			
**Pt4 (S1-S9)**	Duration (s)	100	300	57	36	124	83	128	84	52		
	Avg. RRI (s)	0.64	0.75	1.33	1.72	1.75	3.83	8.11	1.2	1.92		
	Avg. HR (bpm)	93	80	45	35	34	16	7	50	31		
	ECG features	ST-depression, SDNN=3.7	ST-depression, with gradual decline of HR	ST-depression, with further decline of HR	Sinus bradycardia with complete heart block	Sinus arrest, with junctional escape	Sinus arrest, with junctional escape; more profound bradycardia	15s asystole; bradycardia with marked QT elongation	Agonal rhythm with bizarre QRS morphology	Agonal rhythms with bizarre QRS morphology		

#: averaged (Avg.) time interval (seconds) between two adjacent heartbeats (R-peaks)
*: averaged (Avg.) heartrate (HR), in beats per minute (bpm)
$: standard deviation of all normal-to-normal intervals (SDNN; ms)
^: atrioventricular (AV)

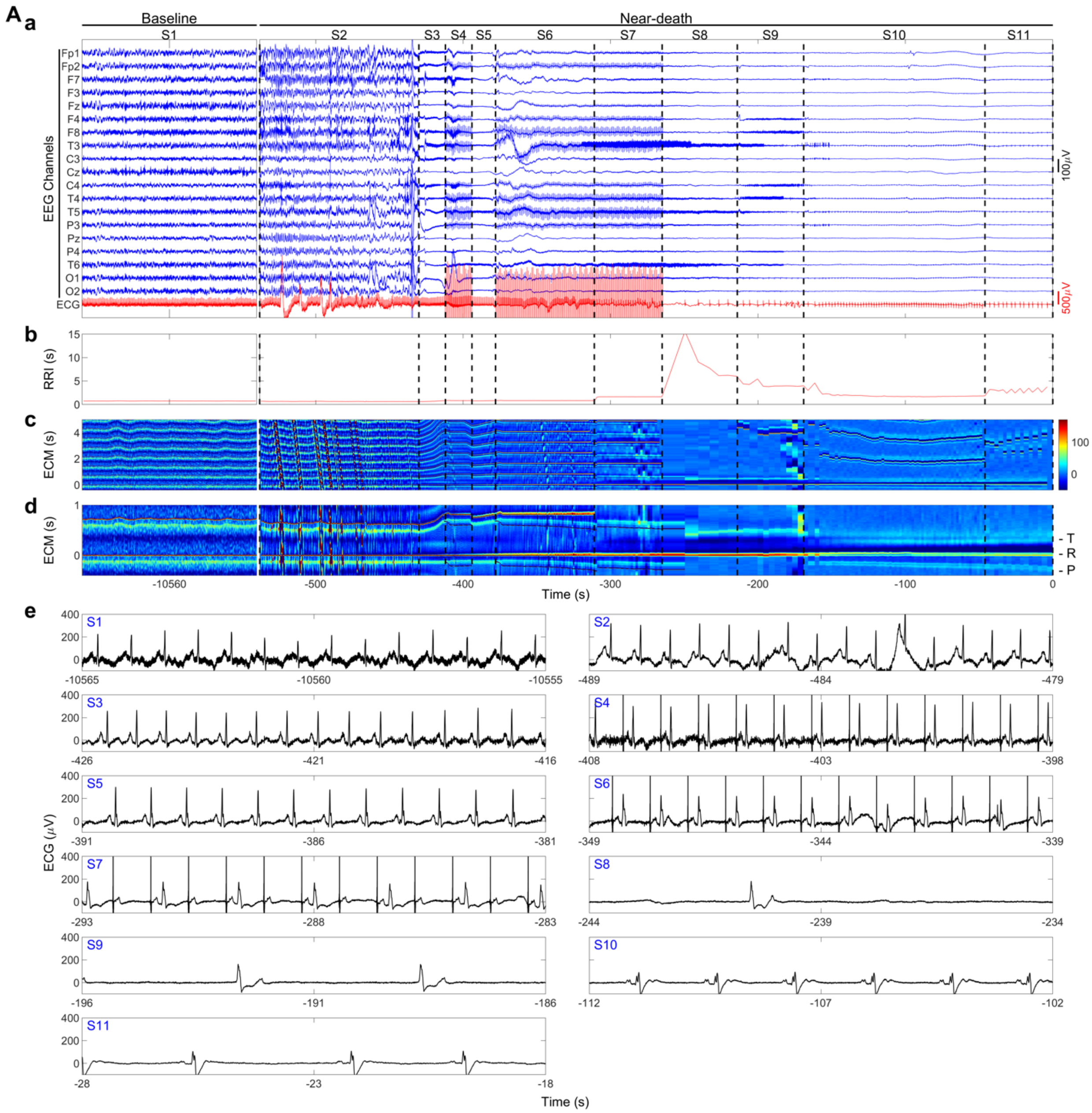

Fig. S1A. Identification of near-death stages in Pt1. a, EEG raw signal from 19 channels (Notch filtered; blue tracings) and ECG signal (red tracing) from the comatose baseline (S1) and the dying stages (S2-S11). S2 begins from the start of ventilator removal and ends at the start of EEG suppression. Multiple episodes of motion artifacts, visible as ECG baseline wandering in S2, were due to physical contact of the medical staff with the patient immediately following extubation (wiping patient's month/face, adjusting body/head position, touching patient's hands, etc.) and confirmed by survey of recorded video. S3 starts from the EEG suppression and ends at the start of external atrial pacemaking, whereas S4 represents the first episode of pacemaking. The pacemaker was automatically turned off (S5) and restarted from S6. S7 starts when rapid heartrate drop was seen which ended when pacemaker was turned off by clinical staff. S8 denotes the bradycardia period where RR interval (RRI) is longer than 5s. In S9, RRI is below 5s (partial heartrate recovery). S10 saw the reappearance of the P-waves and further recovery of heartrate. S11 ends at the last recorded heartbeat with periodical PAC-like ECG pattern. **b,** Temporal dynamics of RRI (seconds). **c, d**, electrocardiomatrix (ECM) expression of the temporal dynamics of ECG changes with longer (panel c) and shorter (panel d) vertical view. The low heartrate variability (HRV) was evident in S1 and S2 as the two R-peak rows are nearly parallel with each other on ECM. Periods with motion artifacts are evident in S2 on ECM showing abnormal color changes centered around the -500s time point. In S3, liner expansion of RRI [seen as increasing vertical time interval between the two rows of R peaks (warmest color), which represents the declining heartrate]. In S4, RRI as well as PR interval (time interval between P-waves and Q-waves) were maintained at a constant rate. In S5, a further and linear expansion of RRI was detected. In S6, while RRI was maintained at a constant level by the pacemaker, PR interval (PRI)'s linear expansion is evident. During S7, 2:1 second degree AV block appeared in the presence of the pacemaker. Turning off the pacemaker at the end of S7 resulted in a 14s asystole and subsequent partial heartrate recovery during S8 and S9 when P-waves disappeared (so called idioventricular rhythm). In S10 and S11, further recovery was associated with reappearance of P-waves and sinus rhythms, but a linear expansion of PRI. **e,** 10s ECG strip was taken from the middle of each of the 11 stages. Power line contamination in S1 and S4, and baseline wandering due to motion artifacts in S2 were visible from the ECG strips.

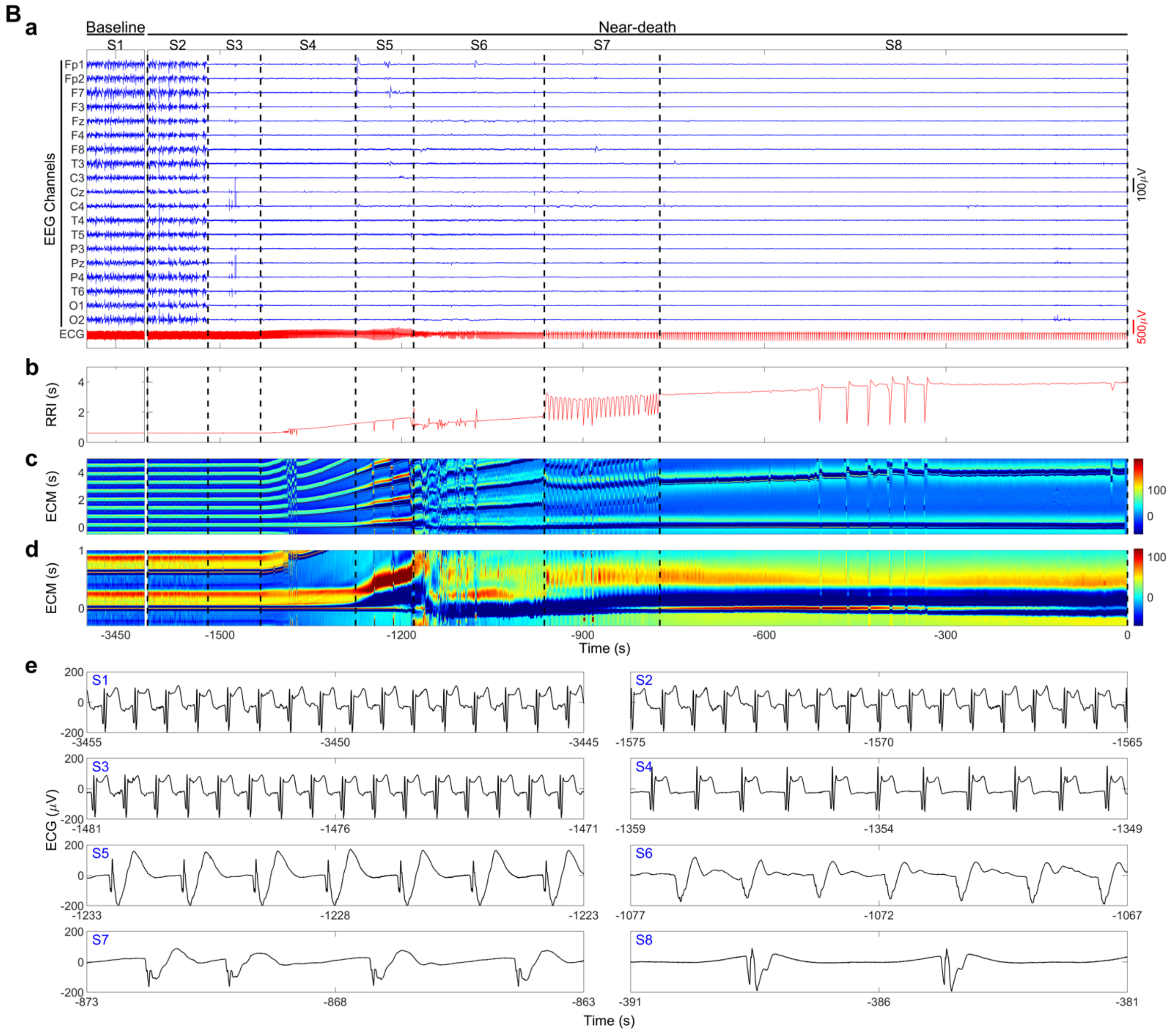

Fig. S1B. Identification of near-death stages in Pt2. a, EEG and ECG raw signals from the comatose baseline (S1) and the dying stages (S2-S8). The patient was supported by ECMO and ventilation in S1, which were turned off at the start of S2. From S3, EEG amplitude was markedly suppressed. **b**, Temporal changes of RRI. RRI was maintained until the end of S3. A linear expansion of RRI was seen in S4 and S5, with occasional interruption. In S6, a brief shortening of RRI was followed by continued lengthening of RRI with rhythmic lengthening and shortening in S7. S8 represents the longest period at the near-death state when RRI was stable with occasional interruption, ending at the last recorded heartbeat. **c, d**, Electrocardiomatrix (ECM) expression of the temporal dynamics of ECG changes with longer (panel c) and shorter (panel d) vertical view. HRV was not detectable in S1-S3, as the two R-peak rows are parallel with each other on ECM. A strikingly high ST-elevation is visually evident during S1-S4 period. A marked increase of T-wave amplitude (warmer color on T-waves) as well as a linear QT elongation were apparent in S5 on ECM (panel d). In S6, an abrupt reduction in QT interval as well as T-wave amplitude was seen with disrupted rhythms. **e,** 10s ECG strip, from the middle of each of the 8 stages, confirm the marked ST-elevation (S1-S4), as evident on ECM.

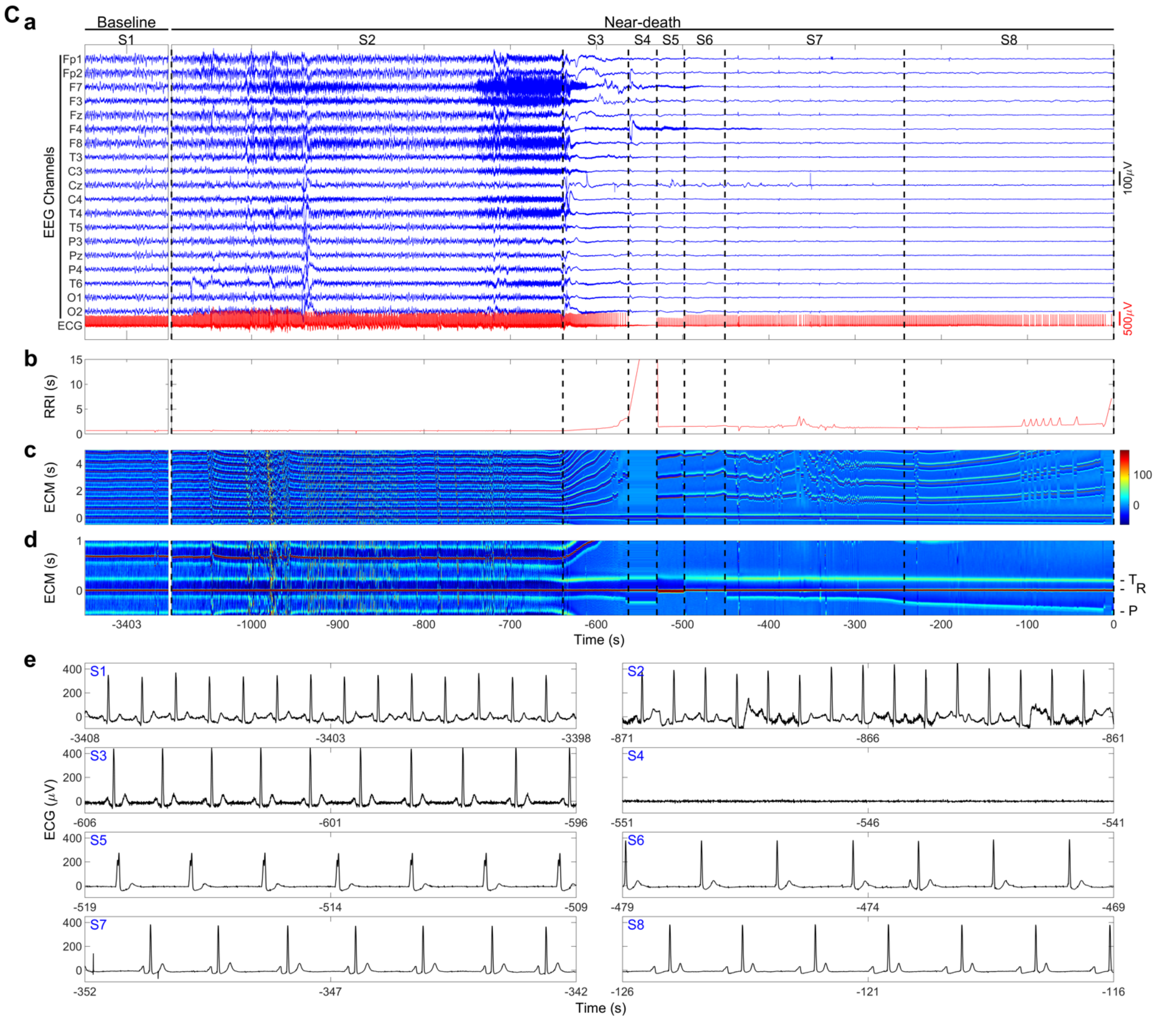

Fig. S1C. Identification of near-death stages in Pt3. a, EEG and ECG raw signals from the comatose baseline (S1) and the near-death stages (S2-S8). Extubation procedure began at the start of S2, and took approximately 60 seconds, during which physical contact between medical staffs and Pt3 was seen on the recorded video footage. EEG suppression, starts at S3, is associated with gradual reduction of heartrate. A 33s of asystole denotes S4. A partial return of heartrate was seen from S4-S8. **b**, Temporal changes of RRI. A linear expansion of RRI was seen in S3. The RRI, though reduced from S1, was seen partially recovered from S5 to S8. **c, d**, ECM view of the dying process. HRV was low at baseline (S1). In S5, heartrate was partially recovered. During this period, R-peak duration increased. P-waves became invisible. In S6, while heartrate was maintained at the same level as in S5, R-peak width returned to a normal level but still without visible P-waves. In S7, while heartrate saw further recovery (shortening of RRI), PR interval lengthening became evident on ECM. The PR-interval continued linear expansion in S8, which ended at the last recorded heartbeat. **e,** Eight 10s ECG strips were from the middle of each of the 8 stages. In S2, ECG baseline appears to show periodical noise that could be due to labored breathing following extubation. In addition, split R-peaks and widening of QRS complex (consistent the ECM pattern in panel d) were seen in S5. Junctional rhythms are evident in S5 and S6. Sinus rhythms returned in S7 and S8, though with increasing PR interval (compare the ECG strips for S7 and S8).

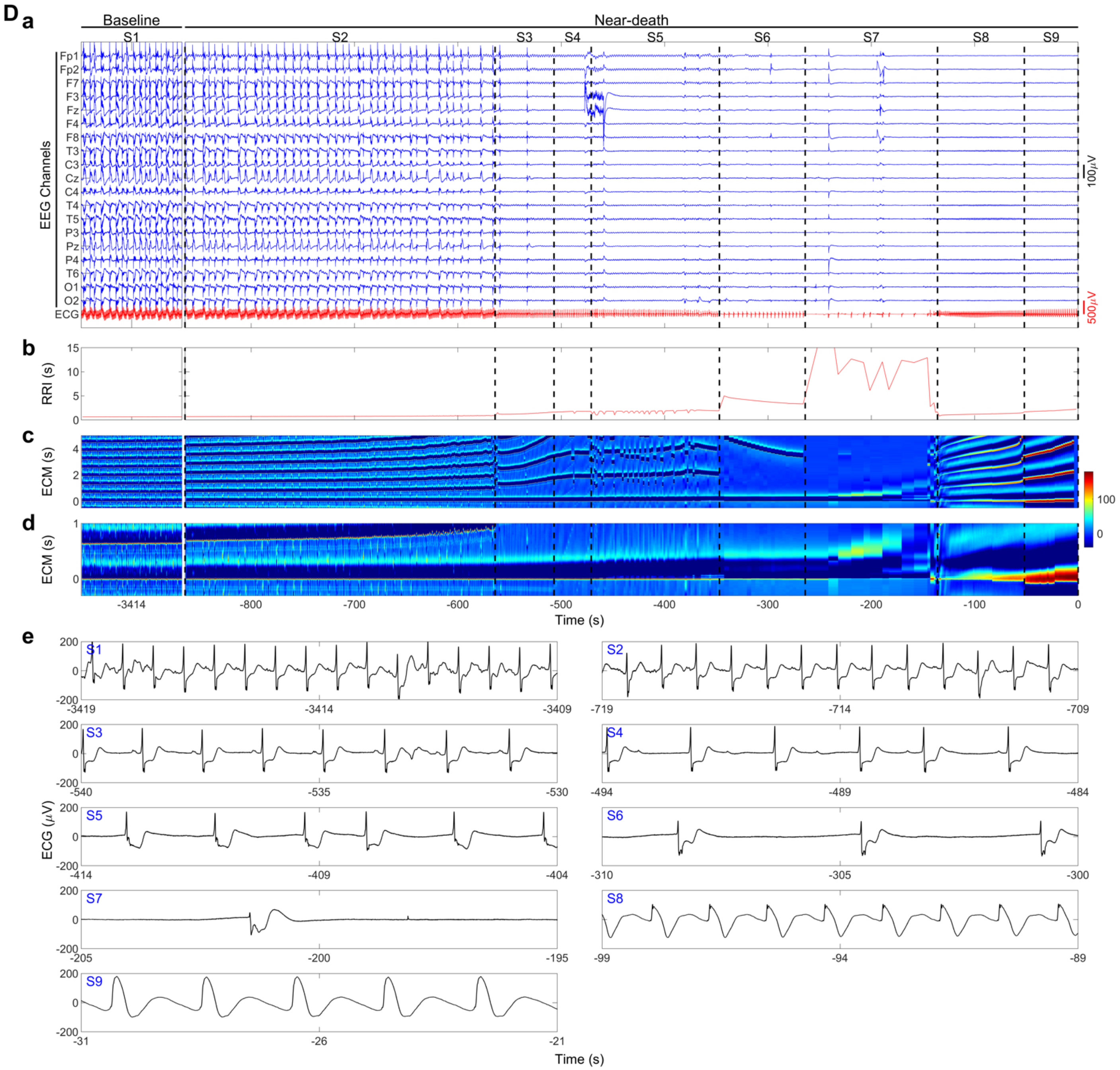

Fig. S1D. Identification of near-death stages in Pt4. a, EEG and ECG raw signals from the comatose baseline (S1) and the near-death stages (S2-S9). EEG signals showed marked burst suppression patterns in S1 and S2, which ended at the start of S3. From S3, EEG amplitude was severely reduced and near flatline. ECG showed several brief episodes of asystole in S7, which showed last second recovery in S8/S9. **b**, Temporal changes of RRI. **c, d**, ECM view of the dying process. HRV was nearly absent at baseline (S1). ST-depression is evident on ECM. Widening of QRS width and increased amplitude of R-peak in S8 and S9 were visually apparent. **e,** Nine 10s ECG strips were from the middle of each of the 8 stages. Morphology of the QRS complex changed markedly in S8 and S9.

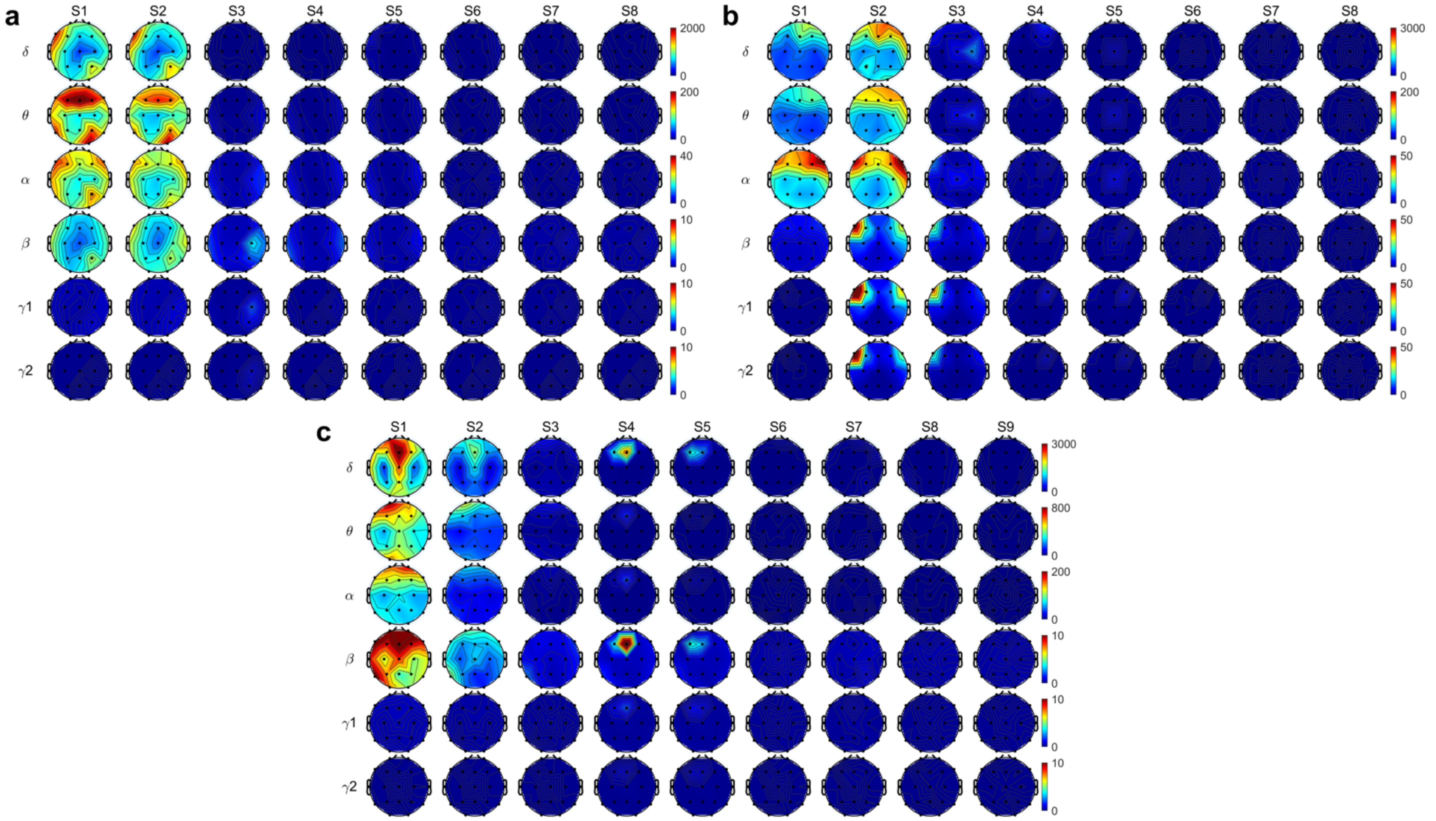

Fig. S2. High frequency oscillations are detectable only in select dying patients. Of the three additional patients included in this study, namely patients 2 (Pt2, **a**), 3 (Pt3, **b**), and 4 (Pt4, **c**), Pt3 (panel **b**) also displayed marked rise of gamma (>25 Hz) powers at near-death (S2 and S3).

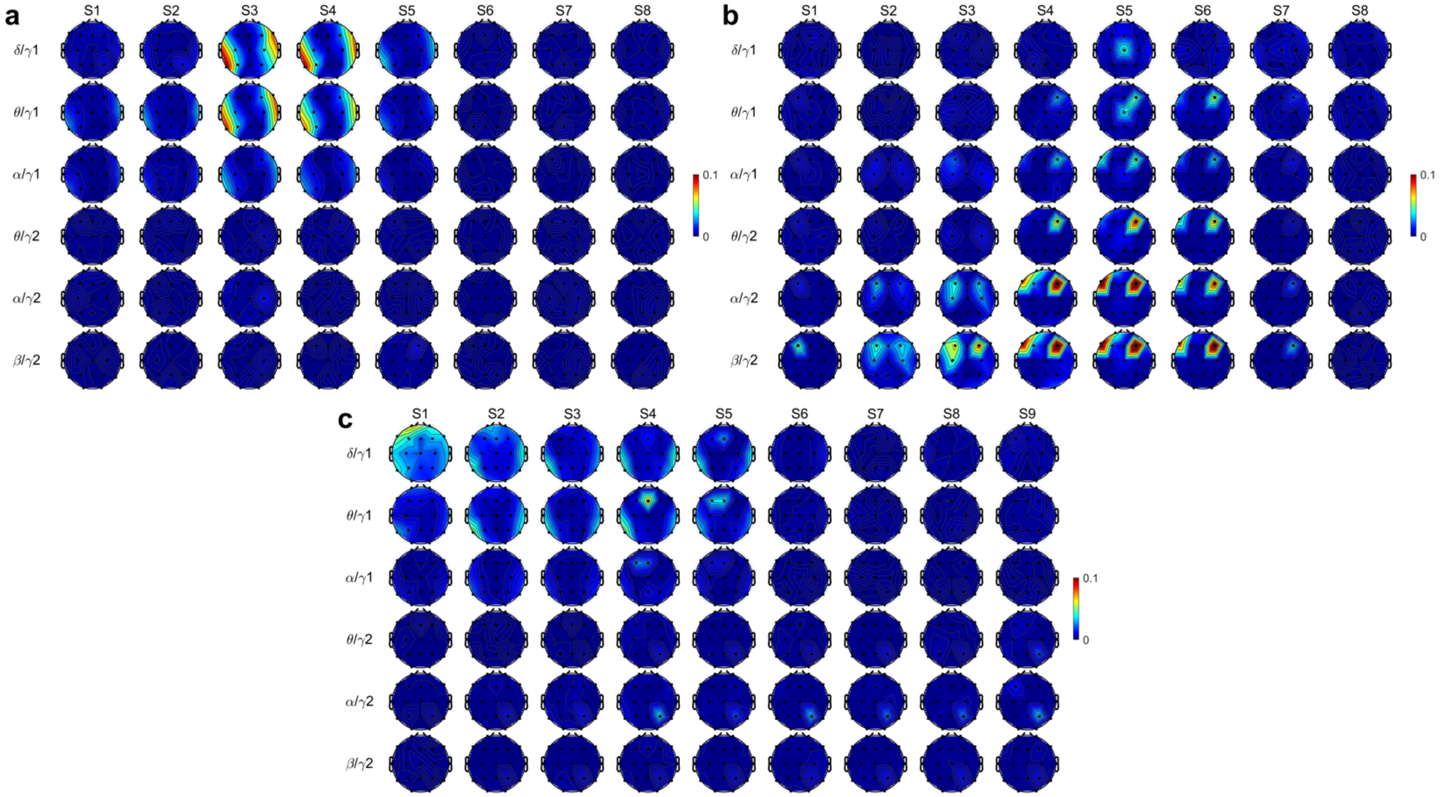

Fig. S3. Phase-amplitude coupling (PAC) of gamma2 amplitude and phase of slower (<25 Hz) oscillations increase is detectable only in subset of the dying patients. Of the three additional patients included in this study, shown in the panels **a** (Pt2), **b** (Pt3), and **c** (Pt4), Pt3 also displayed PAC surge at near-death, with the highest levels seen on beta/gamma2 PAC (panel **b**). Delta/gamma1 and theta/gamma1 PACs, though detectable in all patients (panels **a-c**), are not the focus of this study.

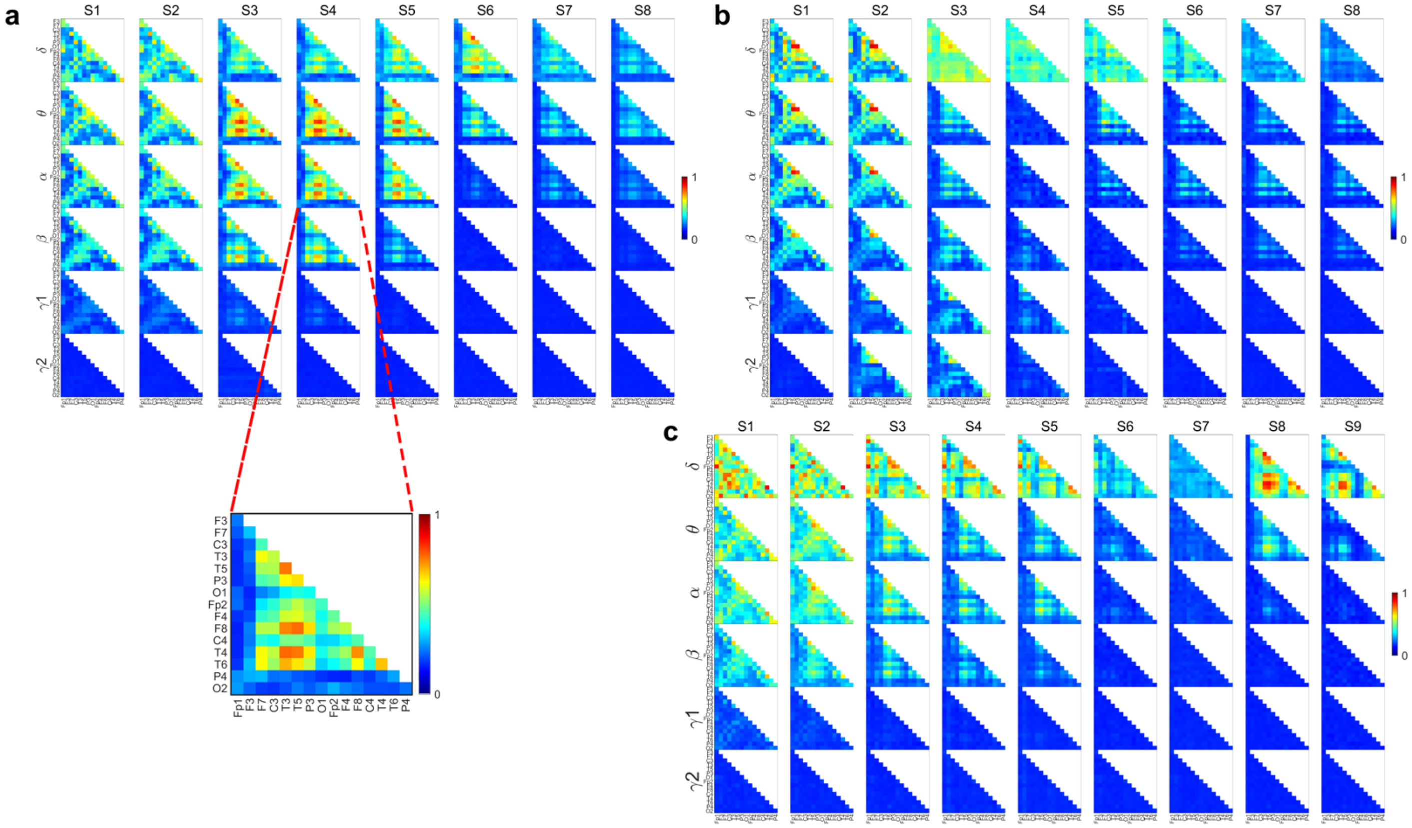

Fig. S4. Surge of functional connectivity is detectable in all patients across multiple frequency bands. a, The rise of coherence across the brain in patient2 (Pt2) is evident at near-death stages (S3-S6), particularly in delta (S6), theta (S3-S5), alpha (S3-S5), and beta (S3 and S4) bands. The enlarged matrix, taken from alpha band in S4, is meant to display the layout of the coherence pairs, with warmer color indicating higher coherence. **b,** Rise of coherence is evident in patient3 (Pt3) in gamma (gamma1 and gamma2) oscillations at near-death (S2, S3). High levels of coherence in slower oscillations (delta-beta) were detected within the left temporo-parietal-occipital (TPO) junctions (T5P3, P3O1, O1T5) at both S1 and S2. **c,** Rise of coherence is evident in patient4 (Pt4) at late near-death stages of S8 and S9 in delta frequency band.

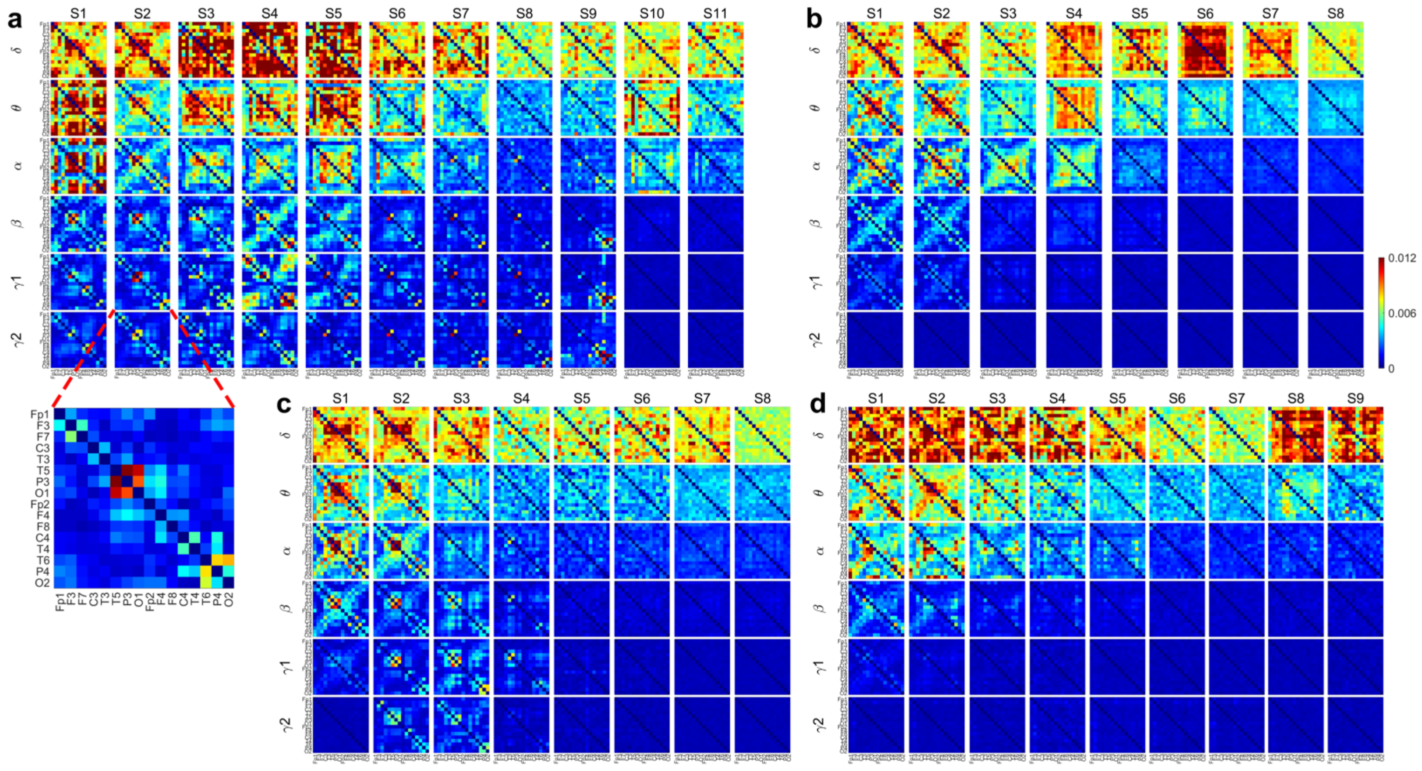

Fig. S5. Effective connectivity is elevated at near-death at multiple frequency bands in all patients. a, In patient1 (Pt1), the near-death rise is prominent in gamma (gamma1 at S2 and S9; gamma2 in S2-S9) frequencies. The direction of connectivity is from the electrodes listed on the Y-axis to those on the X-axis. **b,** Rise of effective connectivity was evident in patient2 (Pt2) at near-death for delta (S4-S7) and theta (S4) frequency bands. **c,** For patient3 (Pt3), the increased effective connectivity is evident for gamma1 and gamma2 bands at near-death (S2-S4). **d,** The rise of effective connectivity appears in delta bands in S8 and S9 in patient4 (Pt4). The rise of effective connectivity in gamma oscillations is not detected at near-death for Pt2 and Pt4.

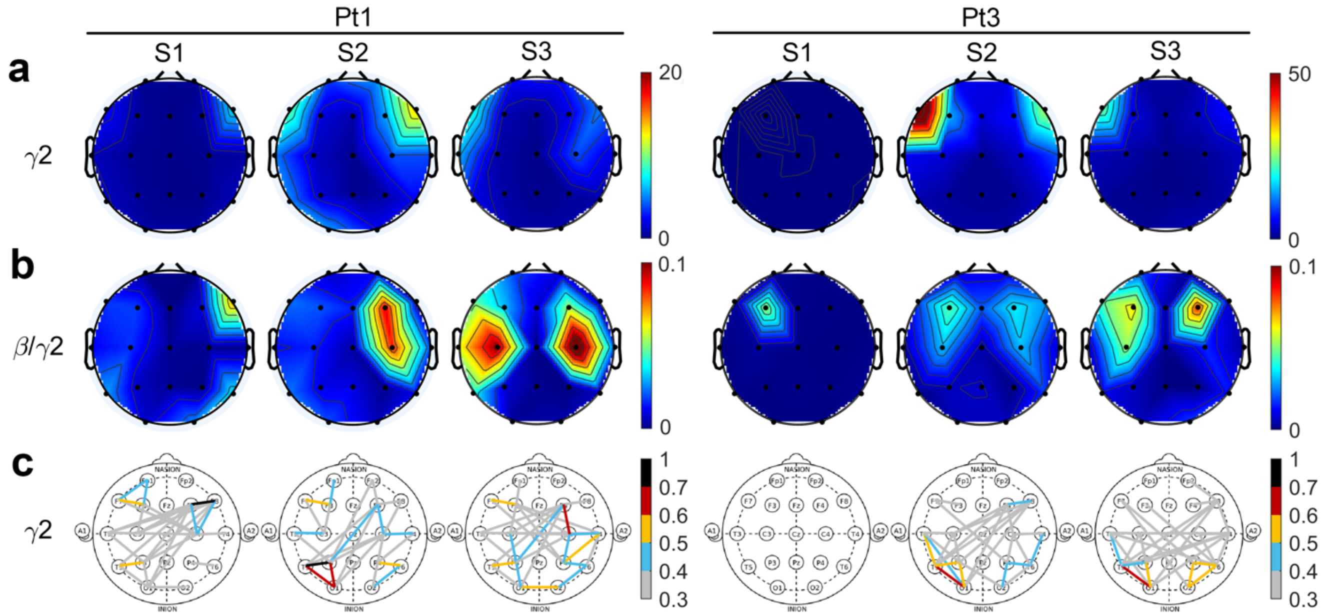

Fig. S6. Temporal dynamics of cortical gamma2 activity during the early dying phase in Pt1 (left) and Pt3 (right). Gamma2 power (panel a; from Fig. 1b for Pt1 and Fig. S2b for Pt3), PAC between beta and gamma2 bands (panel b; from Fig. 2b for Pt1 and Fig. S3b for Pt3), and functional connectivity at gamma2 frequency band (panel c: gamma2 coherence in Fig. 4b for Pt1 and in Fig. S4b for Pt3 during S1-S3, re-expressed here as a topoplot for ease of temporal comparison) are temporally (S1-S3) aligned.

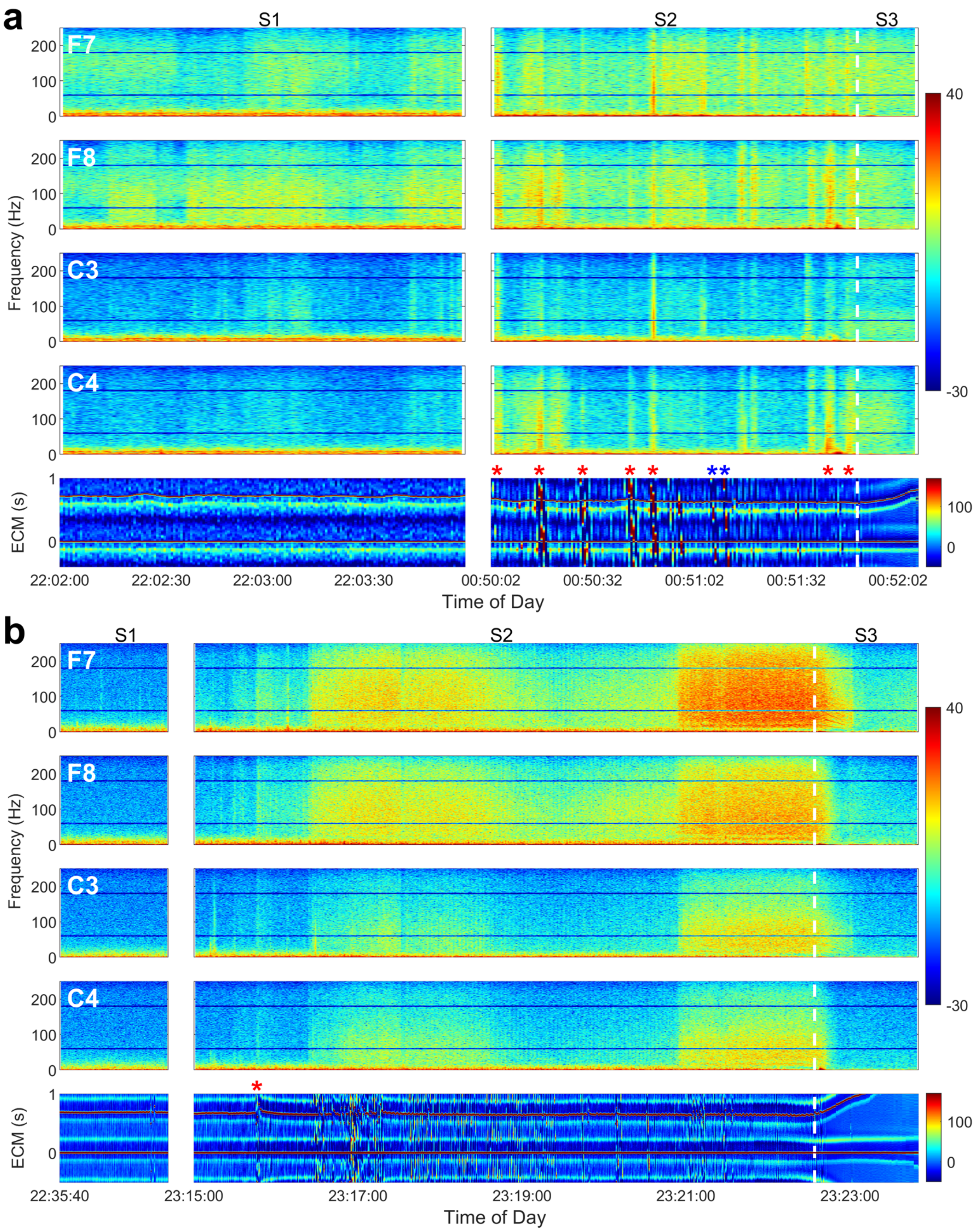

Fig. S7. Power spectrograms from 4 EEG channels and ECM during S1-S3 from Pt1 (a) and Pt3 (b). Red asterisks denote the position of motion artefacts when staff touched the patient's body (head, face, month) during extubation, whereas the blue asterisks indicate the time when staff adjusted the patient's cloths or blankets, as observed on recorded video footage.

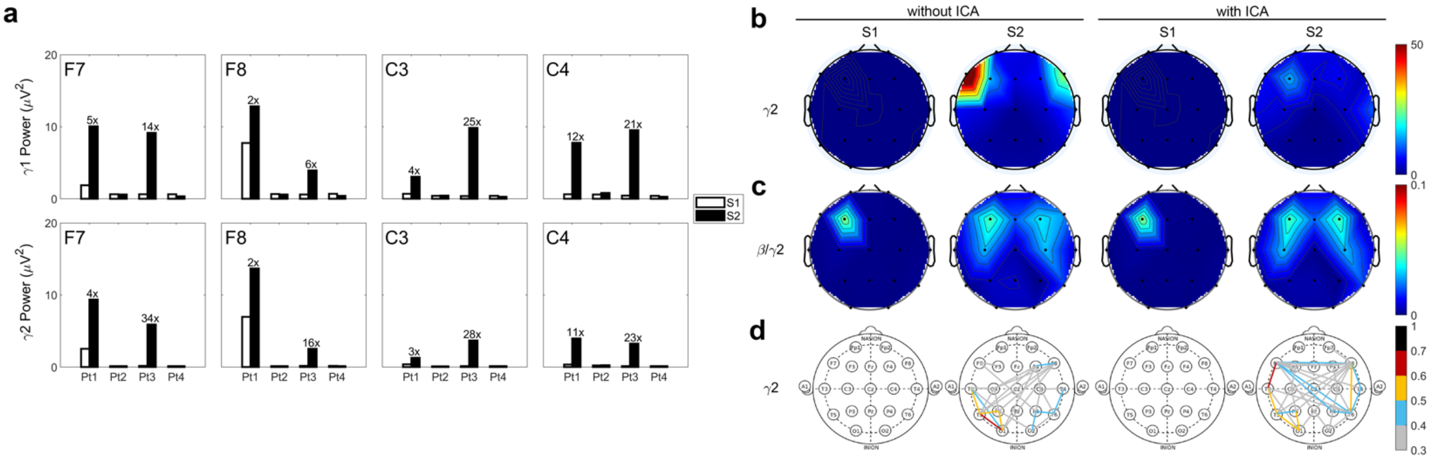

Fig. S8. Independent component analysis (ICA) of Pt3 during the S2 period. a, Gamma power in four cortical regions (F7, F8, C3, and C4) in the 4 patients at baseline (S1) and after the termination of breathing support in S2, following the removal of muscle components identified in S2 for Pt3. **b-d**, reanalysis of the Pt3's S1 and S2 data without (left two columns) and with (right two columns) application of the ICA method. Gamma2 power (panel **b**), PAC between beta and gamma2 bands (panel **c**), and functional connectivity at gamma2 frequency band (panel **d**) are temporally (S1-S2) aligned.
